# Molecular Basis for Vulnerability to Mitochondrial and Oxidative Stress in a Neuroendocrine CRI-G1 Cell Line

**DOI:** 10.1371/journal.pone.0014485

**Published:** 2011-01-04

**Authors:** Natasha Chandiramani, Xianhong Wang, Marta Margeta

**Affiliations:** Department of Pathology, University of California San Francisco, San Francisco, California, United States of America; University of Bremen, Germany

## Abstract

**Background:**

Many age-associated disorders (including diabetes, cancer, and neurodegenerative diseases) are linked to mitochondrial dysfunction, which leads to impaired cellular bioenergetics and increased oxidative stress. However, it is not known what genetic and molecular pathways underlie differential vulnerability to mitochondrial dysfunction observed among different cell types.

**Methodology/Principal Findings:**

Starting with an insulinoma cell line as a model for a neuronal/endocrine cell type, we isolated a novel subclonal line (named CRI-G1-RS) that was more susceptible to cell death induced by mitochondrial respiratory chain inhibitors than the parental CRI-G1 line (renamed CRI-G1-RR for clarity). Compared to parental RR cells, RS cells were also more vulnerable to direct oxidative stress, but equally vulnerable to mitochondrial uncoupling and less vulnerable to protein kinase inhibition-induced apoptosis. Thus, differential vulnerability to mitochondrial toxins between these two cell types likely reflects differences in their ability to handle metabolically generated reactive oxygen species rather than differences in ATP production/utilization or in downstream apoptotic machinery. Genome-wide gene expression analysis and follow-up biochemical studies revealed that, in this experimental system, increased vulnerability to mitochondrial and oxidative stress was associated with (1) inhibition of ARE/Nrf2/Keap1 antioxidant pathway; (2) decreased expression of antioxidant and phase I/II conjugation enzymes, most of which are Nrf2 transcriptional targets; (3) increased expression of molecular chaperones, many of which are also considered Nrf2 transcriptional targets; (4) increased expression of β cell-specific genes and transcription factors that specify/maintain β cell fate; and (5) reconstitution of glucose-stimulated insulin secretion.

**Conclusions/Significance:**

The molecular profile presented here will enable identification of individual genes or gene clusters that shape vulnerability to mitochondrial dysfunction and thus represent potential therapeutic targets for diabetes and neurodegenerative diseases. In addition, the newly identified CRI-G1-RS cell line represents a new experimental model for investigating how endogenous antioxidants affect glucose sensing and insulin release by pancreatic β cells.

## Introduction

Mitochondrial dysfunction has multifactorial etiology. In rare but severe inherited mitochondrial disorders (which typically present in childhood or early adulthood and affect metabolically active organs such as the brain, heart, liver, and skeletal muscle), mitochondrial dysfunction is a result of germline mutations in nuclear or mitochondrial DNA. In contrast, accumulation of somatic mitochondrial DNA mutations that accompanies aging or an impairment in mitochondrial function caused by metabolic and environmental factors are thought to contribute to the pathogenesis of many common age-associated disorders including diabetes, cancer, and neurodegeneration [1]. In diabetes, impaired mitochondrial metabolism contributes to insulin resistance observed in peripheral tissues [2], [3], in part through increase in the level of reactive oxygen species (ROS) [4]. There is also accumulating evidence that mitochondrial dysfunction blunts glucose-stimulated insulin secretion (GSIS) in pancreatic β cells. In a β cell line, for example, GSIS is inhibited following depletion of native mitochondrial DNA and can be restored by repopulation of cybrid cells with foreign mitochondrial DNA [5]. Similarly, GSIS impairment caused by a partial loss of the pancreatic transcription factor PDX-1 (heterozygosity of which leads to a form of the maturity-onset diabetes of the young) is mediated by changes in the mitochondrial gene expression [6], [7]. While it is currently not understood how mitochondrial dysfunction leads to the GSIS impairment, changes in the ROS metabolism are an important candidate because mitochondrially produced ROS act as a signal both in the hypothalamic glucose sensing [8] and the β cell GSIS [9].

Given that mitochondrial dysfunction plays an important role in the pathogenesis of both diabetes and neurodegeneration, it is perhaps not surprising that epidemiologic studies have shown a link between the two [10]. For example, it is well established that patients with diabetes have an increased risk of dementia and/or Alzheimer's disease (reviewed in [11]). To further dissect the relationship between these disorders, recent studies have stratified patients into subcategories depending on the severity and duration of diabetes on one hand and the subtype of dementia on the other. In one such prospective cohort study, diabetes overall was associated with increased risk of vascular dementia, while borderline and undiagnosed diabetes were associated with increased risk of Alzheimer's disease [12]. In another prospective study, higher risk of Alzheimer's disease was associated not with low insulin sensitivity but with low early insulin response to oral glucose challenge, a measure of insulin release [13]; this finding, in particular, raises the possibility that the link between the two diseases reflects an intracellular signaling defect in a pathway common to neurons and pancreatic β cells. A link between diabetes and Parkinson's disease is also suggested by the epidemiologic literature, but its nature is currently unclear: case control studies performed thus far mostly showed that diabetes was associated with a decreased risk of Parkinson's disease, while prospective cohort studies either showed that diabetes was associated with an increased risk of Parkinson's disease or that there was no association between the two diseases ([14]; [15] and references therein). The discrepancy between these results raises the possibility that – like Alzheimer's disease – Parkinson's disease is not associated with the diabetic state per se, but with some underlying metabolic or signaling abnormality that is shared between neurons and β cells. While additional epidemiologic studies with better patient stratification are required to clarify the connections between diabetes and Parkinson's disease, together these studies underscore the underlying biological similarities between neurons and β cells and highlight the importance of identifying shared signaling pathways that modulate susceptibility to mitochondrial dysfunction in neuroendocrine cells.

Here, two subclones of an insulinoma cell line that differ in the susceptibility to mitochondrial and oxidative stressors were used to identify gene expression changes associated with vulnerability to mitochondrial dysfunction in the neuronal/endocrine cell type.

## Results

### Isolation of a CRI-G1 cell line subclone that shows increased vulnerability to mitochondrial and oxidative stress

**Figure 1:**
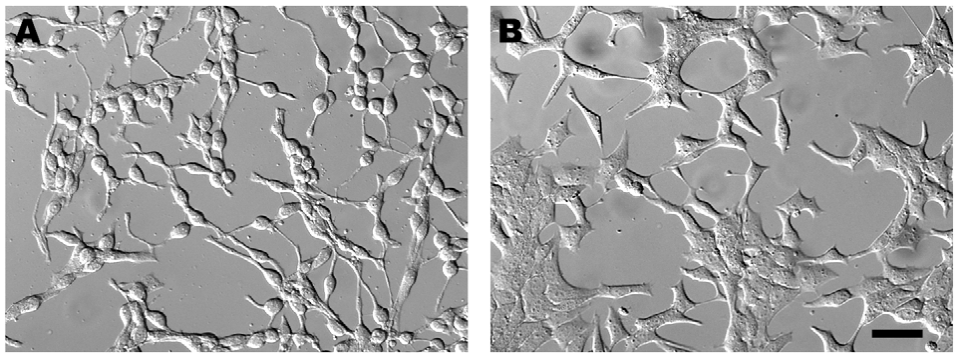
Morphology of CRI-G1 cell lines. **A**. Parental CRI-G1 cells are rounded, have slender processes, and grow in clumps and ribbons rather than forming an epithelial monolayer. In subsequent text and figures, this phenotype is termed CRI-G1-RR (for explanation of the nomenclature, see *Results* section.) **B**. Prolonged culturing at high density conditions leads to a preponderance of cells with surface-adherent, epithelioid appearance that grow in clusters or islands. In subsequent text and figures, this phenotype is termed CRI-G1-RS. Images were acquired by differential interference contrast (DIC) microscopy of living cultures; scale bar, 50 µM.

**Figure 2:**
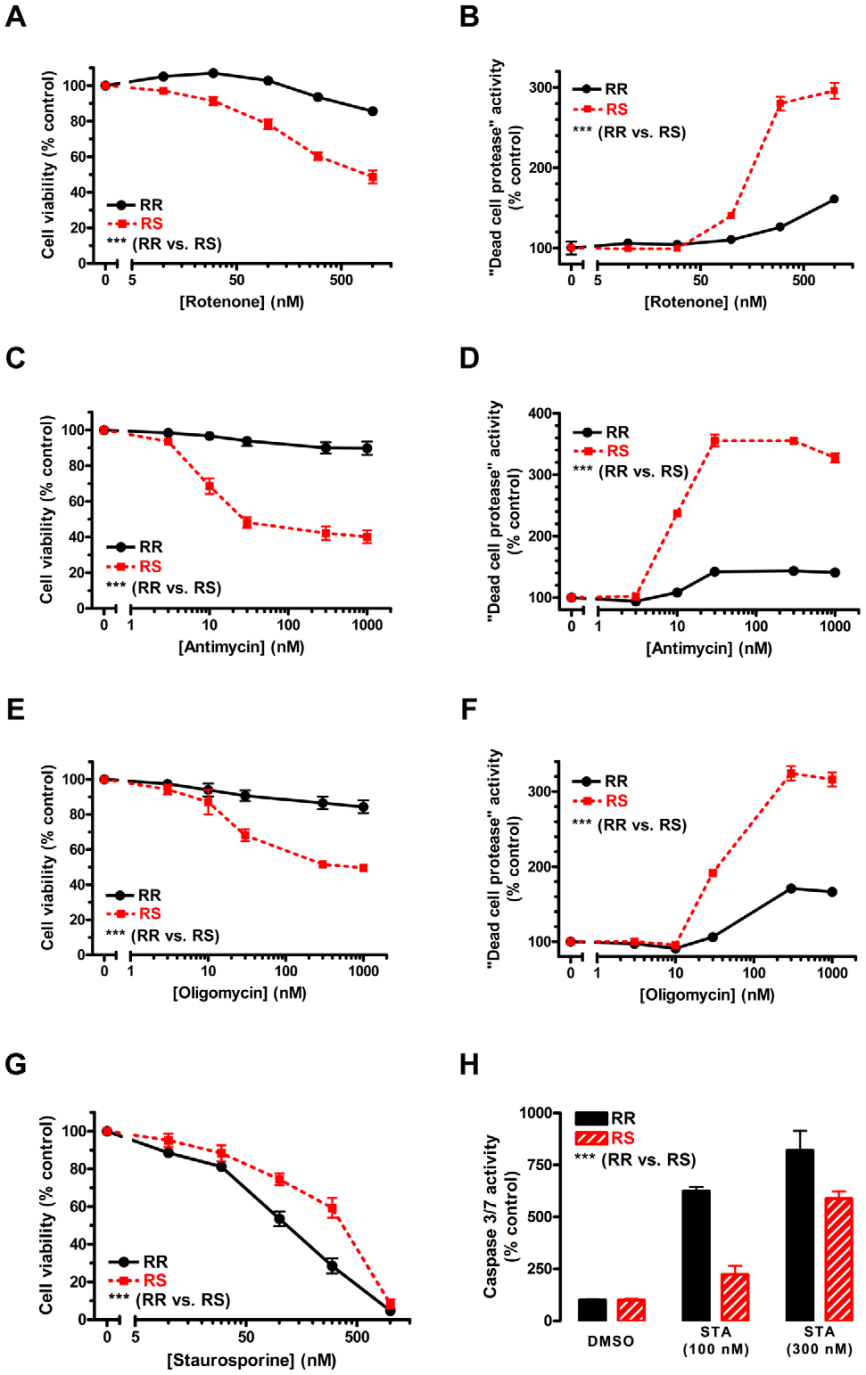
Differential vulnerability to mitochondrial inhibition and staurosporine-induced apoptosis. CRI-G1-RS cells are more vulnerable to cell death induced by mitochondrial respiratory chain inhibitors rotenone (panels A and B), antimycin (panels C and D), and oligomycin (panels E and F), but less vulnerable to apoptosis induced by protein kinase inhibitor staurosporine (panels G and H). Cell viability (panels A, C, E and G) was measured by CellTiter 96 AQ_ueous_ One Solution Cell Proliferation Assay (“MTS assay”), a variant of the classic MTT assay; averaged data from 3–6 repeat experiments are shown in each panel. Cell death (panels B, D, and F) was determined by CytoTox-Glo™ Assay, which measures the activity of a “dead-cell” protease released into the media from membrane-compromised cells; a representative of at least 3 repeat experiments is shown in each panel (n = 4 in each experiment). Caspase 3/7 activity, an indicator of apoptosis, was measured by Caspase-Glo™ 3/7 Assay (panel H); a representative of 3 repeat experiments is shown (n = 4 in each experiment). Although MTS assay is based on mitochondrial metabolism and thus could be directly affected by mitochondrial inhibitors, there was a good correspondence between viability and cell death assay results for all compounds tested. Statistical significance was determined by two-way ANOVA, which showed highly significant effect of both treatment and cell type in all conditions tested. Data are plotted as mean ± S.E.M.; ***, p<0.001.

CRI-G1 is one of four cell lines isolated from a transplantable Cambridge Rat Islet cell tumor in 1985 [16]. CRI-G1 cells are rounded, have slender processes, and grow in clumps and ribbons rather than forming an epithelial monolayer ([16] and Fig. 1A). When maintained in culture for extended periods of time, however, CRI-G1 cells flatten and become more epithelioid in appearance (Fig. 1B); this transition is stochastic in nature, but can be facilitated by growing cells at high densities. The differences between the two CRI-G1 cell subtypes are not restricted to morphology, however: compared to the parental clone, which is resistant to cell death induced by mitochondrial complex I inhibitor rotenone, the novel CRI-G1 cell subclone (which we cryopreserved as a separate cell line after one of the stochastic transition events) is highly susceptible to this mitochondrial toxin, with 51.4±3.6% loss of viability and ∼3 fold increase in release of an intracellular protease (marker of cell death) following an overnight treatment with a 1 µM rotenone (Figs. 2A and B). [In this and all subsequent figures, the original clone is termed CRI-G1-RR (for Rotenone-Resistant) and the novel subclone CRI-G1-RS (for Rotenone-Susceptible).] Similar results were seen with longer treatments (up to 5 days) and higher doses of rotenone (up to 10 µM; not shown).

To determine whether CRI-G1-RS cell line is more susceptible to cell death in general, we tested two additional mitochondrial toxins (complex III inhibitor antimycin and complex V inhibitor oligomycin) as well as staurosporine, a protein kinase inhibitor and potent apoptosis inducer. Interestingly, CRI-G1-RS cells were more susceptible to both antimycin- (Figs. 2C and D) and oligomycin-induced cell death (Figs. 2E and 2F), but less susceptible to staurosporine-induced apoptosis (Figs. 2G and H). Of the three mitochondrial inhibitors tested, antimycin was most toxic to CRI-G1-RS cells (maximal viability loss of 59.9±3.7%), but no mitochondrial toxin led to 100% CRI-G1-RS cell loss regardless of the length of the treatment (up to 5 days) or the dose used (data not shown). In contrast, the maximal dose of staurosporine (1 µM) was completely toxic to both cell types, with lower susceptibility of CRI-G1-RS cells apparent only in the mid-range part of the dose-response curve (Fig. 2G). Consistent with the cell viability data, the increase in caspase 3/7 activity was greater in CRI-G1-RR than in CRI-G1-RS cells at these mid-range doses (100 and 300 nM; Fig. 2H). The three mitochondrial inhibitors led to only minimal activation of caspase 3/7 in both cell types (data not shown), suggesting that in this experimental system cell death induced by mitochondrial inhibition is largely non-apoptotic in nature.

**Figure 3:**
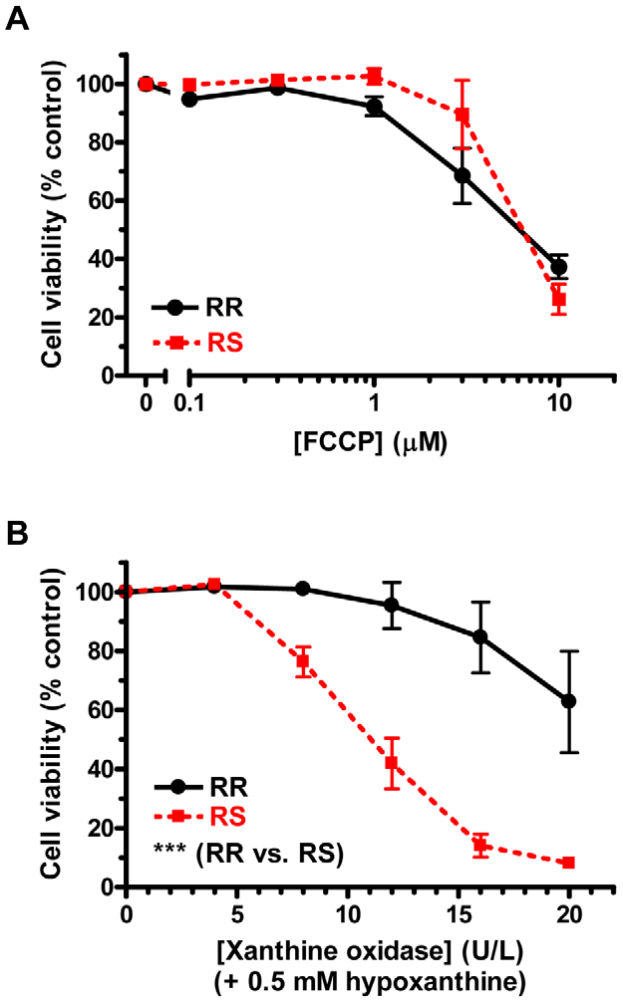
Differential vulnerability to ATP depletion and oxidative stress. **A**. CRI-G1-RR and RS cells are equally vulnerable to mitochondrial uncoupler FCCP, which leads to ATP depletion. **B**. CRI-G1-RS cells are more vulnerable than RR cells to oxidative stress induced by co-application of hypoxanthine and xanthine oxidase. Cell viability was measured by CellTiter 96 AQ_ueous_ One Solution Cell Proliferation Assay, a variant of the classic MTT assay; an average of 3 repeat experiments is shown in each panel. Statistical significance was determined by two-way ANOVA; all data are plotted as mean ± S.E.M. ***, p<0.001.

In general, mitochondrial inhibition leads to (1) a decrease in ATP synthesis, eventually resulting in ATP depletion and (2) an increase in production of ROS, which are the byproduct of the respiratory chain activity; the relative magnitude of each effect depends on the specific respiratory chain complex targeted by the inhibitor and the inhibitor concentration used [17]. To determine whether increased vulnerability to mitochondrial inhibition seen in CRI-G1-RS cell line is due to a diminished ability to cope with ATP depletion or to increased vulnerability to oxidative stress, we examined the susceptibility of both cell types to cell death induced by mitochondrial uncoupler FCCP (which leads to mitochondrial depolarization and ATP depletion in the absence of significant ROS production [18], [19]) or by ROS-generating hypoxanthine/xanthine oxidase (HX/XO) system (which results in oxidative stress with no direct effect on ATP production [20]). FCCP was equally toxic to both cell types at all doses tested (Fig. 3A), suggesting that there was no difference between the two cell lines in their ability to produce (or use) ATP. Consistent with this finding, the total mitochondrial mass (determined by immunoblotting for mitochondrial proteins Tom20 and cytochrome c; Supplemental Figs. S1-A and S1-B) did not differ between CRI-G1-RR and -RS cells. (Interestingly, the level of CoxIV – a subunit of mitochondrial complex IV – was 2.24 fold higher in RS cells relative to RR cells [n = 8, p<0.0001], suggesting an increase in expression of respiratory chain proteins without change in the overall number of mitochondria in this cell type; Supplemental Fig. S1-C.) In contrast, CRI-G1-RS cells were significantly more susceptible to oxidative stress generated by HX/XO treatment (Fig 3B). This effect was dose dependent, with the greatest difference observed at 16 U/L of XO (in the presence of 0.5 mM HX): following overnight treatment, the residual viability at this dose – the second largest tested – was 84.6±12.0% for RR cells but only 14.1±3.9% for RS cells.

**Figure 4:**
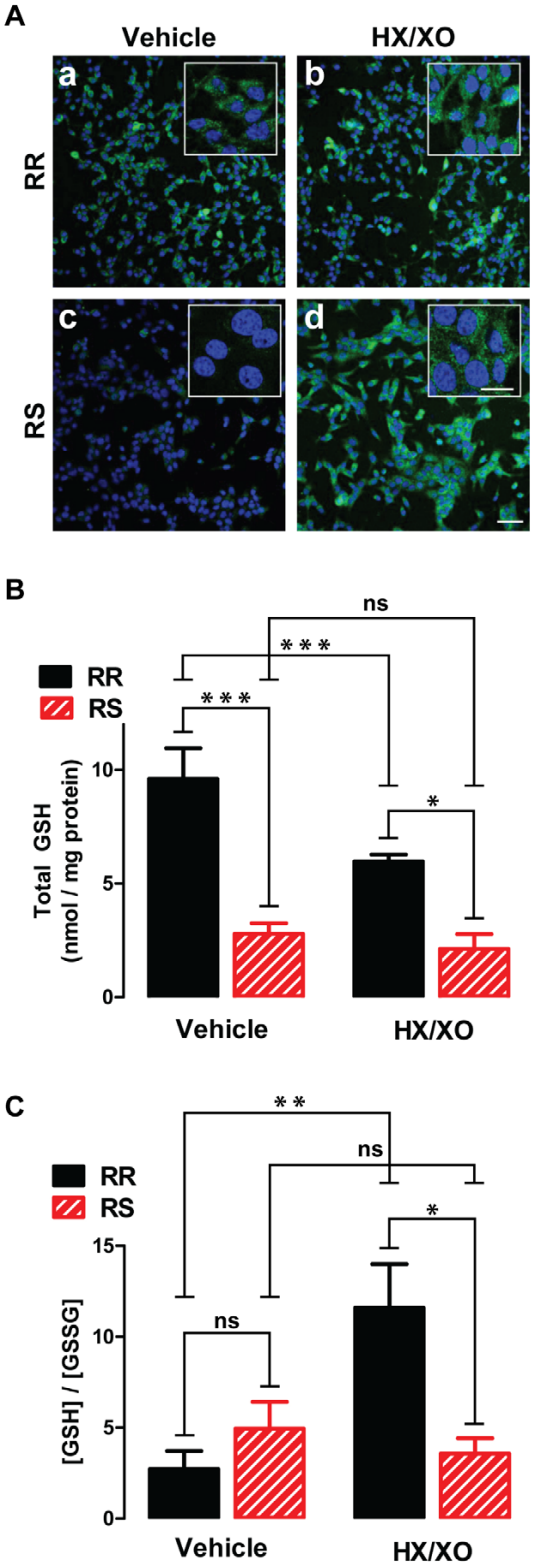
Differential handling of ROS at baseline and following oxidative challenge. **A**. Immunofluorescence staining for lipid peroxidation marker 4-HNE (green) shows that baseline ROS level is higher in RR cells (**a**) than in RS cells (**b**). After treatment with HX/XO, an increase in 4-HNE staining is seen in RS cells (**d**) but not in RR cells (**c**). Nuclei are visualized by DAPI counter stain (blue). Images were acquired by confocal microscopy; scale bars: 50 µm, main panels; 20 µm, insets. A representative of >3 independent experiments is shown. **B**. Total GSH level is higher in RR than RS cells both at baseline and after HX/XO treatment; the oxidative challenge significantly decreased total GSH in RR cells only. **C**. GSH/GSSG ratio is not significantly different between RR and RS cells at baseline. Following HX/XO treatment, it increased in RR cells but did not change in RS cells, resulting in a significant difference between the two cell types. In all experiments, HX/XO treatment consisted of 0.5 mM HX and 16 U/L XO and was applied for 1 h. In B and C, statistical significance was determined by two-way ANOVA followed by ad-hoc Bonferroni post-tests (n = 3–4); all data are plotted as mean ± S.E.M. *, p<0.05; ***, p<0.001.

To further evaluate the differences in susceptibility to oxidative stress between RR and RS cells, 1 h treatment with 0.5 mM HX/16 U/L of XO (or vehicle) was followed by immunostaining for 4-hydroxynonenal (4-HNE), a lipid peroxidation product and a marker of the oxidative stress ([21]; Fig. 4A), or by measurement of glutathione (GSH) and glutathione disulfide (GSSG) levels (Figs. 4B, 4C and S2). RR cells showed significant 4-HNE immunoreactivity at baseline (Fig. 4A-a), but no increase in staining following the HX/XO treatment (Fig. 4A-b). In contrast, baseline 4-HNE immunoreactivity in RS cells was barely detectable (Fig. 4A-c), but staining intensity strongly increased following 1 h exposure to HX/XO (Fig. 4A-d). The concentration of total GSH (Fig. 4B) was higher in RR than in RS cells both at baseline (9.6±1.4 vs. 2.8±0.5 nmol/mg protein; n = 4, p<0.001) and after HX/XO treatment (6.0±0.3 vs. 2.1±0.7 nmol/mg protein; n = 3, p<0.05; Fig. 4B); interestingly, oxidant treatment resulted in a statistically significant decrease in the total GSH concentration in RR cells only (p<0.001). The GSH/GSSG ratio, which is an indicator of the cellular redox potential, did not significantly differ between RR and RS cells at baseline (2.7±1.0 vs. 4.9±1.5; n = 4). Following the HX/XO treatment, the GSH/GSSG ratio did not significantly change in RS cells (from 4.9±1.5 to 3.6±0.9; n = 3); in RR cells it paradoxically increased (from 2.7±1.0 to 11.6±2.4; n = 4, p<0.01), resulting in a statistically significant difference between the two cell types under oxidizing conditions (p<0.05). The increase in the GSH/GSSG ratio observed in RR cells after oxidant exposure is a result of a decrease in the concentration of GSSG rather than an increase in the concentration of GSH (Supplemental Fig. S2); this is likely due to an increase in the GSSG efflux that can complicate interpretation of GSH/GSSG ratio under oxidizing conditions [22]. Taken together, these results indicate that, compared to the parental CRI-G1-RR cells, CRI-G1-RS cells are in a relatively reduced state at baseline, but are more vulnerable to injury and cell death when exposed to oxidative stress. In addition, the data suggest that increased vulnerability of CRI-G1-RS cells to mitochondrial inhibition is primarily a result of the lesser capacity to handle metabolically generated ROS.

### Expression profiling

**Figure 5:**
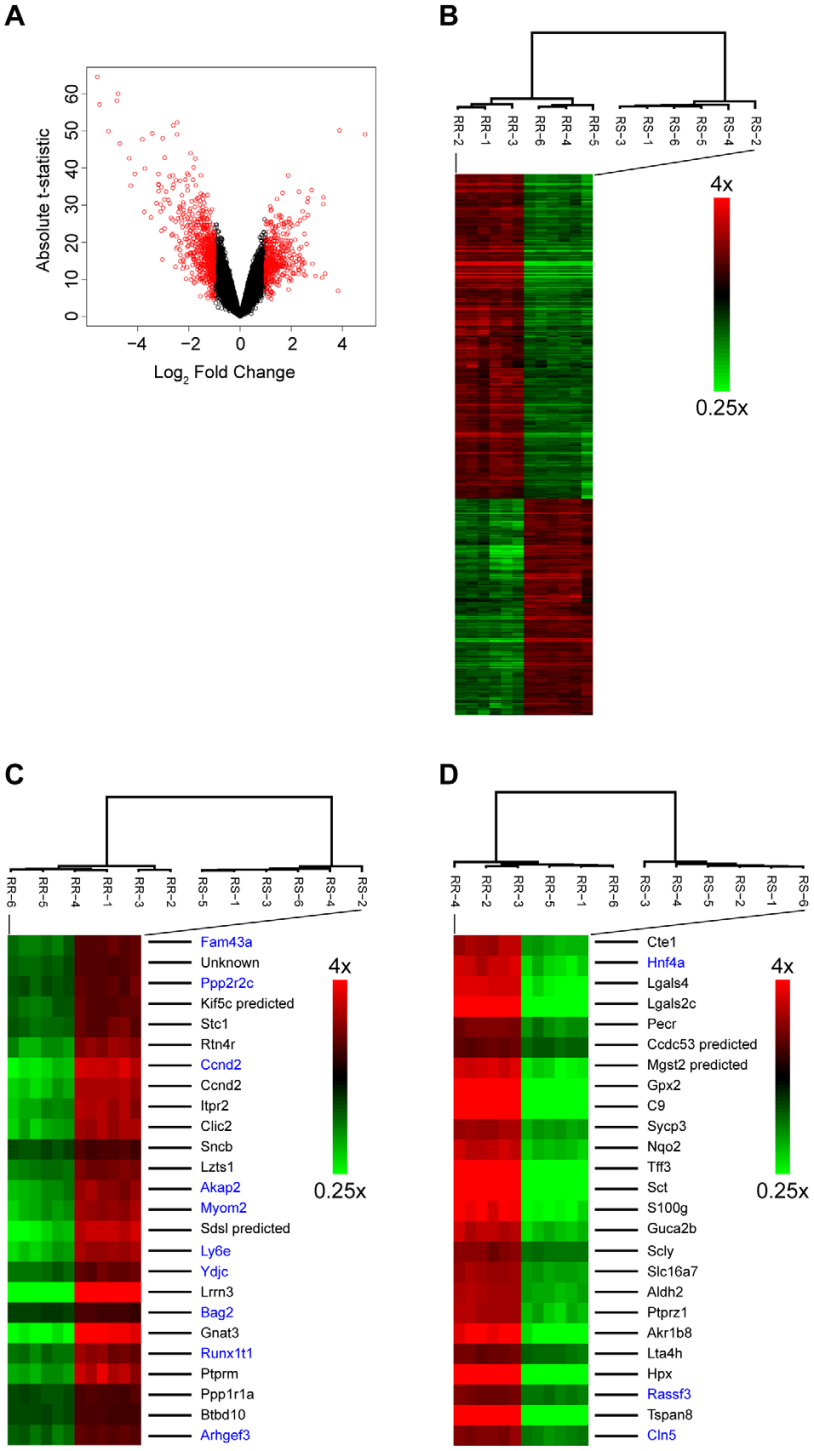
Analysis of gene expression data. **A**. The volcano plot shows that there are many significant genes with high fold change; significant genes (probes with FDR adjusted p-values<0.05) with differential expression ≥2 fold are marked in red. **B–D**. Unsupervised hierarchical clustering shows that independent biological replicates of each cell type group together (high expressing genes, red; low expressing genes, green). The analyses were performed using 999 probesets with differential expression ≥2 fold (panel B), top 25 genes upregulated in RS cells (by p-value; panel C), and top 25 genes downregulated in RS cells (also by p-value; panel D). In panels C and D, black font is used for gene symbols identified by the Affymetrix array profile and blue font for gene symbols identified by manual BLAST search with Affymetrix source sequences. Unknown gene in panel C corresponds to Affymetrix probe 1385716_at (source sequence AW522166).

CRI-G1-RR and RS cell lines are closely related, but show large differences in susceptibility to mitochondrial and oxidative stressors; this enabled us to use genome-wide gene expression analysis to uncover a molecular profile associated with vulnerability to mitochondrial dysfunction. Biotinylated cRNA probes (six independent biological replicates for each cell type) were hybridized to rat Affymetrix 230 2.0 arrays, which contain 31100 oligonucleotide probes representing 14318 unique genes. The statistical analysis showed that 12872 probes, corresponding to 5861 unique genes, showed statistically significant differential expression (FDR adjusted p-values<0.05). A much smaller subset of 999 probes (corresponding to 550 unique genes) showed a large difference in expression (≥2 fold); out of these 550 genes, 216 were upregulated (log_2_fold change from 1 to 4.89) and 334 downregulated (log_2_fold change from −1 to −5.57) in oxidative stress-vulnerable CRI-G1-RS cells (Fig. 5A). Unsupervised hierarchical clustering was performed using 999 probes with differential expression ≥2 fold (Fig. 5B), top 25 genes upregulated in RS cells (by p-value; Fig. 5C), and top 25 genes downregulated in RS cells (also by p-value; Fig. 5D). In all three analyses, independent biological replicates of each cell type clustered together, indicating that phenotypic differences we identified correspond to specific transcriptional profiles. (The same was true when analysis was performed using all probes on the array; data not shown.)

Twenty genes from biologically interesting pathways (discussed further in the next section) were chosen for validation of microarray data by qRT-PCR; of these 20 genes, 10 were downregulated (log_2_fold change range -0.27 to −5.50) and 8 upregulated (log_2_fold change range 0.40 to 1.26) in RS cells, while 2 showed no statistically significant change in expression between the two cell types (Table 1). The quantitative correlation between the microarray and qRT-PCR data was much better for downregulated genes (r^2^ = 0.96, p<0.0001; Supplemental Fig. S3-A) than for upregulated genes (r^2^ = 0.11, p = 0.59; Supplemental Fig. S3-B). Given that independent biological replicates were used for each method, these differences could reflect the pathway-specific accumulation of additional gene expression changes. Alternatively, there may be inherent differences between the two methods that are unevenly distributed across the gene expression spectrum. Nonetheless, the overall correspondence between the two methods was good, with 9 of 10 downregulated genes and 8 of 8 upregulated genes showing statistically significant differential expression and the same direction of expression changes in both experimental paradigms (Table 1); this degree of correspondence is within the range generally described in the literature.

**Table 1:** qRT-PCR validation of microarray data for selected genes.

Gene Symbol	Gene Name	Array Probe ID	Array log_2_FldChg	Array FDR-adjusted p-value	qRT-PCR log_2_FldChg	qRT-PCR p-value
Gpx2	Glutathione peroxidase 2	1374070_at	−5.4946	4.17E-15	−5.4842	<0.0001
Nqo2	NAD(P)H dehydrogenase, quinone 2	1374959_at	−3.0212	1.87E-14	−3.4518	<0.0001
Ephx1	Epoxide hydrolase 1, microsomal	1387669_a_at	−2.3098	1.29E-11	−3.1377	<0.0001
Txnrd1*	Thioredoxin reductase 1	1386958_at	−1.8186	4.98E-09	−2.1232	<0.0001
Nqo1	NAD(P)H dehydrogenase, quinone 1	1387599_a_at	−1.7330	1.30E-08	−2.0396	<0.0001
Gclc*	Glutamate-cysteine ligase, catalytic subunit	1372523_at	−1.3981	1.75E-10	−1.5696	<0.0001
Ucp2	Uncoupling protein 2 (mitochondrial, proton carrier)	1368669_at	−1.1069	4.75E-09	−0.7164	0.0011
Nfe2l2	Nuclear factor, erythroid derived 2	1367826_at	−0.4808	1.04E-04	−0.2784	0.3640
Park7	Parkinson disease (autosomal recessive, early onset) 7	1368653_a_at	−0.4464	5.87E-07	−0.5447	0.0047
Gclm	Glutamate cysteine ligase, modifier subunit	1370030_at	−0.2666	4.14E-03	−0.5451	0.0035
Nkx6-1	NK6 homeobox 1	1368998_at	1.2551	6.21E-09	3.1688	0.0003
Pax4	Paired box 4	1370140_a_at	1.5244	3.52E-09	1.8654	0.0009
Pdx1	Pancreatic and duodenal homeobox 1	1369516_at	1.1104	9.58E-09	1.0633	0.0019
Ins2	Insulin 2	1370077_at	1.0955	1.51E-06	not detectable in RR samples	n.a.
Neurod1	Neurogenic differentiation 1	1387288_at	0.8884	5.07E-09	1.6602	0.0001
Keap1	Kelch-like ECH-associated protein 1	1370066_at	0.4625	2.46E-07	0.7266	0.0004
Ins1	Insulin 1	1387815_at	0.4069	1.63E-05	3.7082	<0.0001
Gck	Glucokinase	1387312_a_at	0.4007	3.34E-05	4.3504	0.0003
Txn1	Thioredoxin 1	1398839_at	0.0446	0.3041	−0.0122	0.9246
Me1	Malic enzyme 1, NADP(+)-dependent, cytosolic	1370067_at	0.0645	0.4560	0.3880	0.0046

Ins2 mRNA was not detectable in RR samples using qRT-PCR; thus, log_2_fold change and p-value could not be calculated for this gene. For genes marked with *, multiple probes were present on the array; the table lists a probe showing the greatest fold change.

To identify pathways that might underlie increased vulnerability to mitochondrial and oxidative stress in CRI-G1-RS cells, we analyzed gene expression data using GOstat [23] and Ingenuity Pathways Analysis (IPA version 7.6; Ingenuity® Systems, www.ingenuity.com). GOstat analysis, performed on 999 probes that showed ≥2 fold change in expression in either direction, identified 53 significant GO annotations (based on an FDR adjusted p-value cutoff of 0.05; Supplemental Table S1). While these 53 GO annotations included a number of different biological processes, the two most common categories were cell metabolism (9 of 53 annotations; p-value range 8.18×10^−5^ to 0.04) and neuronal differentiation or function (another 9 of 53 annotations; p-value range 8.87×10^−5^ to 0.03). Other potentially interesting GO annotations included ER-nuclear signaling pathway (GO ID: 0006984; p = 0.01); response to stress (GO ID: 0006950; p = 0.03); and unfolded protein response (GO ID: 0030968; p = 0.03). Ingenuity Pathways Analysis was performed on an expanded probe set (3917 probes with FDR-adjusted p-value≤0.001, regardless of the fold change; when multiple probes were present for a single gene, the probe with the smallest p-value was used). The top five *Physiological System Development and Function* gene network clusters identified by this analysis were Endocrine System Development and Function cluster (p-value range 0.0002 to 0.05), Tissue Morphology cluster (p-value range 0.0002 to 0.05), Connective Tissue Development and Function cluster (p-value range 0.002 to 0.05), Nervous System Development and Function cluster (p-value range 0.007 to 0.05), and Skeletal and Muscular System Development and Function cluster (p-value range 0.01 to 0.05). Ingenuity Pathways Analysis can also be used to identify canonical pathways that show statistically significant ratio of differentially expressed genes; the top five *Canonical Pathways* identified by IPA in our dataset were Nrf2-mediated Oxidative Stress Response (p-value = 6.73×10^−5^); FAK (Focal Adhesion Kinase) Signaling (p-value = 2.54×10^−3^); EIF2 (Elongation Initiation Factor 2) Signaling (p-value = 3.84×10^−3^); Integrin Signaling (p-value = 3.94×10^−3^); and Galactose Metabolism (p-value = 4.16×10^−3^). Taken together, these data show that a relatively large number of genes is differentially expressed between CRI-G1-RR and CRI-G1-RS cells and that these genes are involved in a number of distinct biological pathways; gene expression changes that might underlie differential vulnerability to mitochondrial and oxidative stress are discussed in more detail in the next section.

### Expression changes in antioxidant and detoxifying enzyme genes

**Figure 6:**
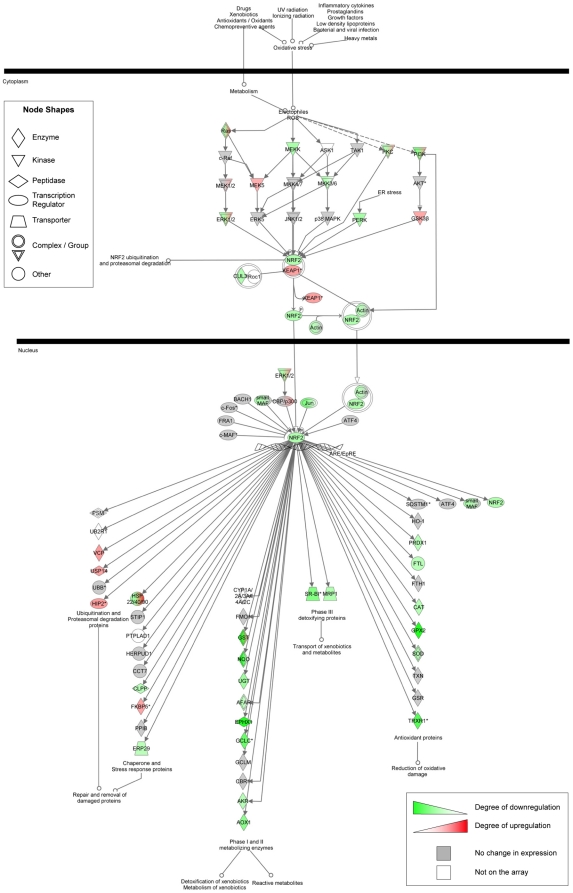
Differential expression of genes in the canonical Nrf2-mediated oxidative stress response pathway. Genes are represented as nodes, biological relationships between two nodes as lines. The intensity of the node color indicates the degree of up- (red) or down- (green) regulation in CRI-G1-RS cells relative to CRI-G1-RR cells; gray node color indicates genes showing no differential expression between RR and RS cells, while white node color marks genes that were not present on the array. Protein complexes/gene groups are indicated by nodes with double outlines; color shading within such a node indicates that genes within the complex/gene group showed different expression changes. The analysis was performed on 3917 probes with FDR-adjusted p-value≤0.001, regardless of the fold change. When multiple probes were present for a single gene, the probe with the smallest p-value was used.

Among top 25 genes downregulated in RS cells (based on p-value; Fig. 5D), three genes (*Gpx2, Mgst2_predicted*, and *Nqo1*) are downstream targets of Nrf2 (nuclear factor-erythroid 2-related factor 2). Nrf2 is a transcription factor that regulates both baseline and inducible expression of a battery of molecular chaperons, antioxidant, and phase I/II conjugation enzymes in a number of organs including the lung, liver and brain [24]; its function in pancreatic β cells, however, is not well understood. The analysis of gene expression changes for the entire Nrf2 pathway, performed by IPA, showed that many Nrf2 target genes (75 of 185) were differentially expressed between CRI-G1-RR and CRI-G1-RS cells (Fig. 6; larger version is provided as Supplemental Fig. S4); for a subset of these genes (*Gpx2*, *Nqo2*, *Ephx1*, *Txnrd1*, *Nqo1*, *Gclc*, and *Gclm*), we confirmed the microarray-identified expression changes by qRT-PCR (Table 1). Interestingly, while Nrf2-regulated antioxidant and detoxifying (phase I/II conjugating) enzymes were downregulated in RS cells, Nrf2-regulated molecular chaperones (including members of Hsp22, 40 and 90 families) were largely upregulated in the same cell type; the basis for this differential regulation of distinct subsets of Nrf2 target genes is currently unclear.

**Figure 7:**
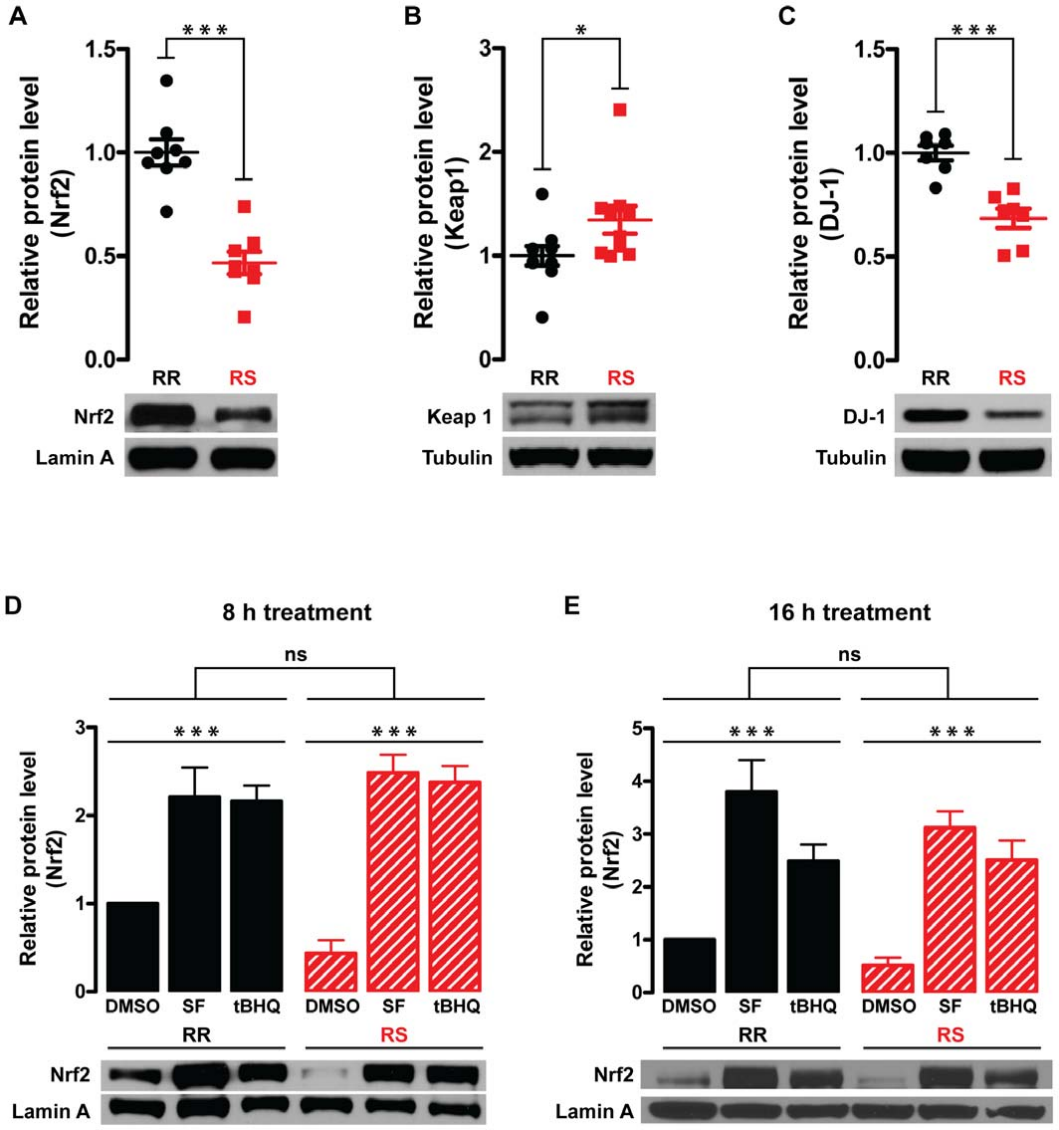
Differential expression of Nrf2, Keap1, and DJ-1. **A**. At baseline, Nrf2 protein level (apparent MW, 77 kDa) in the nuclear fraction is significantly lower in CRI-G1-RS than RR cells. **B**. At baseline, Keap1 protein level (apparent MW, 69 kDa) in the whole cell lysate is significantly higher in CRI-G1-RS than RR cells. **C**. At baseline, DJ-1 protein level (apparent MW, 21 kDa) in the whole cell lysate is significantly lower in CRI-G1-RS than RR cells. **D and E**. Treatment with 5 µM SF or 10 µM tBHQ for 8 h (**D**) or 16 h (**E**) activated Nrf2 pathway to a similar extent in both CRI-G1-RR and RS cells. Apparent MW for lamin A was 68 kDa; for tubulin, 51 kDa. In panels A–C, statistical significance was determined by two-tailed Student's t-test; scatter plots of all data points are shown. In panels D and E, statistical significance was determined by two-way ANOVA; data are plotted as mean ± S.E.M. (n = 5). While there was no statistically significant difference between the two cell types overall, ad-hoc Bonferroni post-tests detected a statistically significant difference between DMSO-treated RR and RS samples (p<0.01), similar to the difference observed with untreated samples (panel A). *, p<0.05; ***, p<0.001.

To further delineate differences in Nrf2 pathway regulation between RR and RS cells, we determined the mRNA and protein expression levels of Nrf2 and its regulator Keap1 (Kelch-like ECH-associated protein 1). Under baseline conditions, Nrf2 is bound to actin cytoskeleton by Keap1, which prevents its translocation into the nucleus and targets it for ubiquitination and proteosomal degradation. In response to oxidative stress, kinase activation, or small molecule activators such as *tert*-butylhydroquinone (tBHQ) and sulforaphane (SF), Nrf2 is stabilized and translocates into the nucleus, where it binds the antioxidant response element (ARE) sequences in the promoter of its target genes and activates their transcription (reviewed in [24], [25]). We also determined mRNA and protein expression levels of DJ-1, which regulates Nrf2 stability in fibroblasts and the lung or lung-derived cell lines [26], [27], but not in neurons or astrocytes [28]. DJ-1 is a redox-sensitive protein [29], [30], mutations in which have been linked to an autosomal recessive form of Parkinson's disease [31]; it has recently been shown to protect β cells from various stress conditions [32]. The microarray data showed a modest but statistically significant decrease in expression of Nrf2 mRNA (*Nfe2l2*; log_2_fold change = −0.48, p-value = 0.0001); however, this finding was not confirmed by qRT-PCR (log_2_fold change = −0.28, p-value = 0.36; Table 1). Nonetheless, the expression of transcriptionally-active Nrf2 protein (assessed by immunoblotting of nuclear fractions with anti-Nrf2 antibody; Fig. 7A) was significantly lower in RS than in RR cells (53% or ∼2 fold decrease; n = 8, p<0.0001). In cytoplasmic fractions, Nrf2 protein level was below detection threshold in either cell type (data not shown). Significant differences in protein expression without major changes in mRNA expression suggest that regulation primarily occurs on the post-translational level. Consistent with this possibility, we found that RR and RS cells expressed different levels of Keap1 and DJ-1. Specifically, Keap1 was significantly upregulated in RS cells both on mRNA (microarray: log_2_fold change = 0.46, p-value = 2.46×10^−7^; qRT-PCR: log_2_fold change = 0.73, p-value = 0.0004; Table 1) and protein levels (35.6% or 1.36 fold increase; n = 10, p<0.05; Fig. 7B). In contrast, the expression of DJ-1/*Park7* mRNA (microarray: log_2_fold change = −0.45, p-value = 5.87×10^−7^; qRT-PCR: log_2_fold change = 0.54, p-value = 0.005; Table 1) and protein (31.5% or 1.46 fold decrease; n = 7, p = 0.0002; Fig. 7C) was significantly decreased in RS cells. Given the reciprocal functions of Keap1 and DJ-1 in the regulation of Nrf2 pathway, both of these changes would be expected to result in destabilization of Nrf2 protein and subsequent downregulation of Nrf2 target genes in RS cells, as observed in the microarray and qRT-PCR datasets. Interestingly, treatment with 5 µM SF or 10 µM tBHQ for either 8 h (Fig. 7D) or 16 h (Fig. 7E) upregulated nuclear Nrf2 expression to the same extent in both RR and RS cells despite the baseline differences in Nrf2 pathway activity between the two cell types. (Visualizing low basal levels of nuclear Nrf2 protein in both cell types required long film exposures, raising a concern that much higher Nrf2 levels in SF- or tBHQ-treated samples were not accurately quantified. To address this possibility, band densitometry was also performed on short exposure films from the same experiments and showed essentially the same results [Supplemental Fig. S5].) Interestingly, tBHQ-induced activation of Nrf2 pathway was comparable to SF-induced activation following 8 h treatment (Figs. 7D and S5-A), but less strong following 16 h treatment (Figs. 7E and S5-B) in both cell types. Taken together, the data indicate that both the overall activity of Nrf2 pathway and expression level of many Nrf2-regulated antioxidant and detoxifying enzymes are decreased in CRI-G1-RS cells; these genetic changes are likely to mediate (or at least contribute to) increased vulnerability to mitochondrial and oxidative stress seen in these cells.

**Figure 8:**
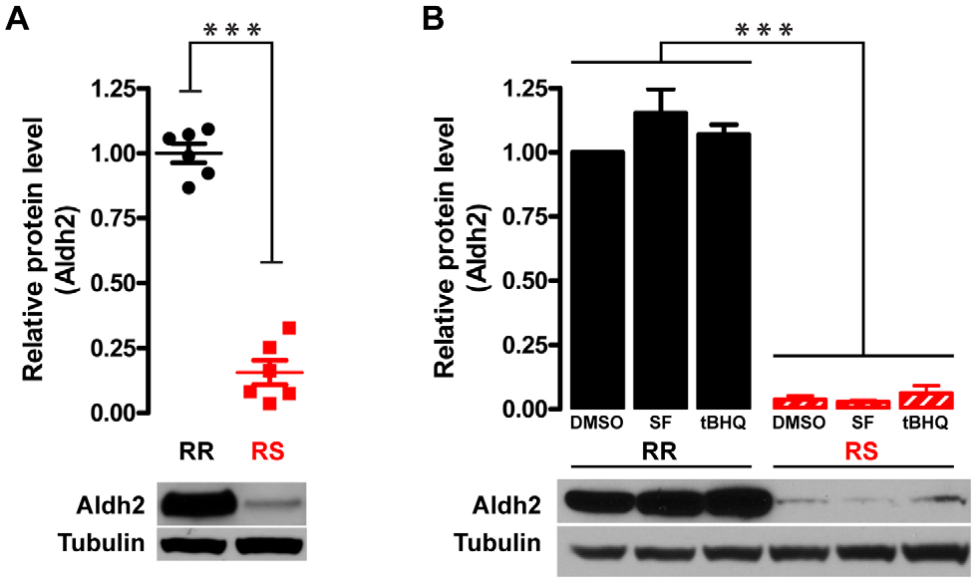
Differential expression of Aldh2 at baseline and following activation of Nrf2 pathway. **A**. At baseline, Aldh2 protein level (apparent MW, 51 kDa) in the whole cell lysate is significantly lower in CRI-G1-RS than in CRI-G1-RR cells. Statistical significance was determined by two-tailed Student's t-test; scatter plot of all data points is shown. B. Aldh2 expression was not affected by 24 h treatment with 5 µM SF or 10 µM tBHQ (two activators of Nrf2 pathway) in either cell type; regardless of the treatment, Aldh2 expression level was significantly lower in CRI-G1-RS than in CRI-G1-RR cells. Statistical significance was determined by two-way ANOVA; data are plotted as mean ± S.E.M. (n = 5). ***, p<0.001.

Another important antioxidant gene that showed high differential expression in the microarray dataset is aldehyde dehydrogenase 2 (*Aldh2*; log_2_fold change = −2.61, p-value = 2.55×10^−18^). Aldh2 plays a key role in the metabolism of ethanol (by catalyzing oxidation of acetaldehyde into acetic acid) and toxic aldehydes such as 4-HNE, which at baseline (but not after oxidant treatment) showed higher immunoreactivity in RR than RS cells (Fig. 4A). In addition, Aldh2 is one of key mediators of the cardiac ischemic preconditioning [33]. Interestingly, a dominant negative mutation of Aldh2 (Aldh2*2), common in Asian populations, leads to alcohol intolerance and increased risk of Alzheimer disease, particularly in ApoE4 carriers [34]; it is not currently known whether it also raises the risk for diabetes or other age and oxidative stress-associated diseases. Expression of Aldh2*2 in cell lines results in increased vulnerability to mitochondrial toxins [35]; in transgenic mice, it leads to age-dependent neurodegeneration accompanied by memory loss [36]. To confirm that observed changes in Aldh2 mRNA levels lead to differences in Aldh2 protein expression in CRI-G1 cells, whole cell lysates from untreated RR and RS cells were immunoblotted with anti-Aldh2 antibody (Fig. 8A). Consistent with mRNA expression data, we found that Aldh2 protein was strongly expressed in RR cells but barely detectable in RS cells (84% or ∼6 fold decrease; n = 6, p<0.0001). While expression of many aldehyde dehydrogenases is regulated by Nrf2 [37], [38], the data for Aldh2 (derived mostly from microarray experiments) are currently equivocal: it was reported that Nrf2 does not regulate either basal or inducible Aldh2 expression [38], that it regulates basal Aldh2 expression only [39], and that it regulates both basal and inducible Aldh2 expression [40]. To determine whether Nrf2 pathway upregulation leads to an increase in Aldh2 expression in the CRI-G1 cell line, we used immunoblotting to measure Aldh2 protein levels following treatment of both RR and RS cells with vehicle (0.2% DMSO), 5 µM SF, or 10 µM tBHQ for 24 hours (Fig. 8B). While Aldh2 protein levels were significantly different between RR and RS cells (two-way ANOVA, n = 5; p<0.0001), as seen with untreated samples (Fig. 8A), neither SF nor tBHQ treatment resulted in Aldh2 upregulation in either cell type (p = 0.28). The same result was seen with shorter (8 h; Supplemental Fig. S6) and longer treatments (up to 48 h; data not shown). Taken together, the data indicate that decreased expression of Aldh2, which most likely is not regulated by Nrf2 in this cell type, represents another potential mechanism for increased vulnerability to mitochondrial dysfunction seen in CRI-G1-RS cells.

### Changes in expression of genes important for β cell differentiation/function and in glucose-sensitive insulin secretion

**Figure 9:**
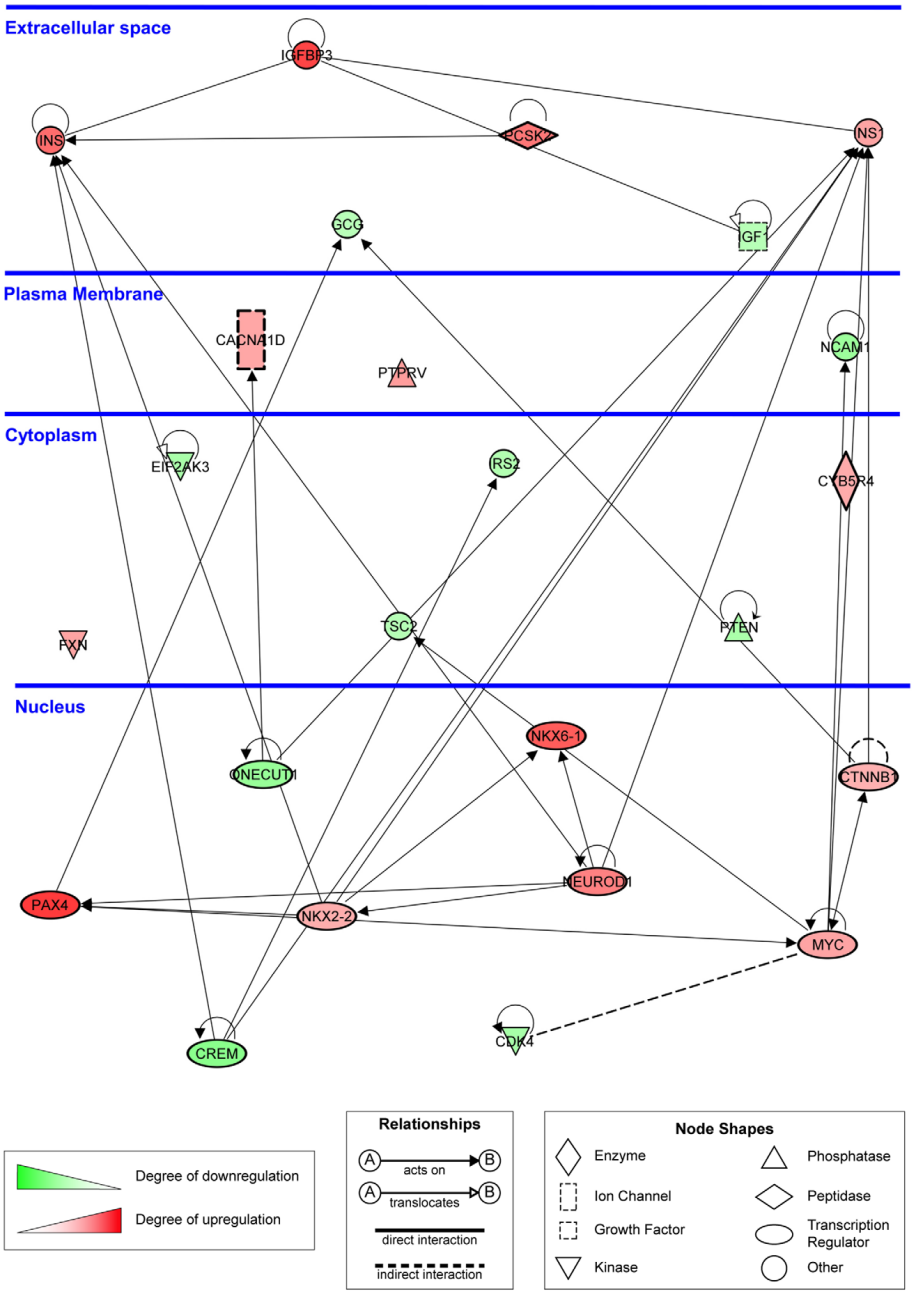
Differential expression of genes important for β cell differentiation and function. Genes are represented as nodes, biological relationships between two nodes as lines. The intensity of the node color indicates the degree of up- (red) or down- (green) regulation in CRI-G1-RS cells relative to CRI-G1-RR cells; the analysis was performed with 3917 probes with FDR-adjusted p-value≤0.001, regardless of the fold change. When multiple probes were present for a single gene, the probe with the smallest p-value was used.

**Figure 10:**
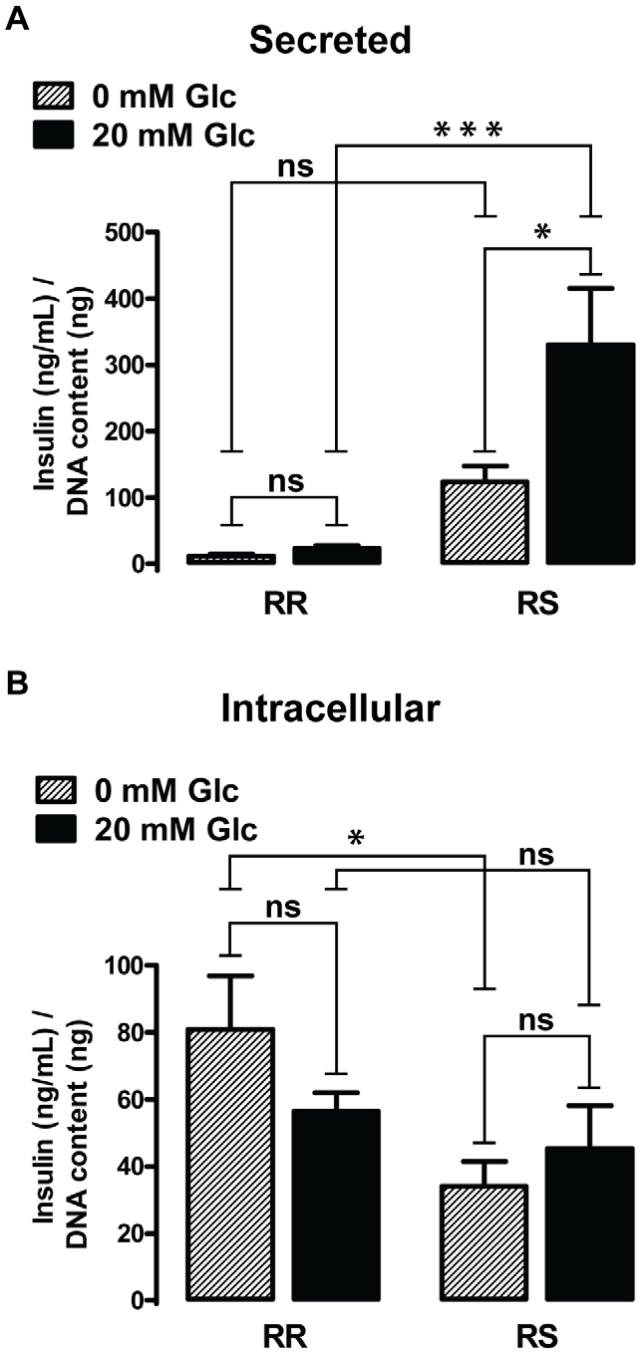
Differences in glucose-induced insulin secretion and intracellular insulin content. **A**. Insulin secretion is induced by 20 mM glucose in RS but not in RR cells. **B**. Intracellular insulin content is modestly but significantly lower in RS than RR cells at 0 mM glucose; there is no difference between the two cell types following the high glucose challenge. Statistical significance was determined by two-way ANOVA followed by ad-hoc Bonferroni post-tests (n = 4); all data are plotted as mean ± S.E.M. *, p<0.05; ***, p<0.001.

The top *Physiological System Development and Function Gene Network* identified by IPA was Endocrine System Development and Function cluster (p-value range 0.0002 to 0.05), which includes networks with overlapping sets of genes, such as Quantity of β Islet Cells, Size of Islet Cells, Proliferation of β Islet Cells, Differentiation of Pancreatic Cells, and Degranulation of β Islet Cells (among others). To illustrate gene expression changes in genes important for β cell differentiation and function, 24 genes that belong to these pathways and show differential expression between CRI-G1-RR and RS cells were compiled in a single IPA-created schematic diagram (Fig. 9; larger version is provided as Supplemental Fig. S7). Interestingly, we found that many transcription factors that are required for specification and/or maintenance of β cell fate, such as Neurod1, Pax4, Pdx1, Nkx6-1, and Nkx2-2 [41], were significantly upregulated in oxidative stress-susceptible CRI-G1-RS cells. Expression of β cell-specific genes, such as insulin 1, insulin 2 and glucokinase [42], was also much higher in CRI-G1-RS cells. (The differential expression of many of these genes was confirmed by qRT-PCR; Table 1.) We thus also examined whether there was a difference in glucose sensitivity and insulin release between oxidant-resistant RR and oxidant-sensitive RS cells (Fig. 10). As described in the original report [16], CR1-G1-RR cells secreted little insulin at baseline (11.3±3.1 ng/mL per 1 ng DNA; n = 4) and did not show significant increase in insulin secretion in response to 20 mM glucose (22.9±4.8 ng/mL per 1 ng DNA; n = 4, p>0.05; Fig. 10A). In contrast, oxidant-susceptible RS cells showed significant increase in insulin secretion following the high glucose stimulus (330.5±85.1 ng/mL vs. 124.0±24.0 ng/mL per 1 ng DNA; n = 4, p<0.05). Interestingly, baseline content of intracellular insulin was slightly lower in RS cells than in RR cells (34.1±7.3 vs. 80.1±16.0 ng/mL per 1 ng DNA respectively; n = 4, p<0.05; Fig. 10B). Given that RS cells express higher level of both Ins1 and Ins2 mRNA (Table 1), the reason for this difference is not clear; one possibility is that chronic secretion induced by maintenance media (which contains 25 mM glucose) resulted in relative insulin depletion that did not completely recover during 3 h incubation in 0 mM glucose (see *Materials and Methods* for details of insulin ELISA). Following stimulation with high glucose, there was no significant difference in the intracellular insulin content between the two cell types (56.6±5.5 ng/mL per 1 ng DNA in RR cells vs. 45.4±12.8 ng/mL per 1 ng DNA in RS cells; n = 4, p>0.05). Taken together, the data raise a possibility that CRI-G1-RS cell line corresponds to a more differentiated β cell phenotype and support the recent hypothesis that increased expression of antioxidant enzymes can lead to glucose resistance [43].

## Discussion

Many age-associated disorders (including diabetes, cancer, and neurodegenerative diseases) are associated with oxidative stress and mitochondrial dysfunction; at least in part, this may reflect accumulation of mitochondrial genome mutations that occurs with aging [1]. Pancreatic β cells express very low levels of antioxidant enzymes [44] and are particularly vulnerable to mitochondrial dysfunction and oxidative stress, which are thought to significantly contribute to pathogenesis of both type 1 and type 2 diabetes [4], [45], [46]. Here, we show that increased vulnerability to mitochondrial dysfunction and oxidative stress in an insulinoma cell line is associated with (1) morphologic change to a flat, surface-adherent phenotype accompanied by gene expression changes in integrin/FAK signaling pathways (Supplemental Figs. S8 and S9); (2) increased expression of β cell-specific genes and transcription factors that specify/maintain β cell fate; (3) restored GSIS; (4) downregulation of ARE/Nrf2/Keap1 antioxidant pathway; (5) decreased expression of antioxidant and phase I/II conjugation enzymes, many of which belong to the Nrf2 pathway; and (6) increased expression of molecular chaperones, many of which, interestingly, also belong to the Nrf2 pathway.

Given the important role of Nrf2 pathway in susceptibility to oxidative stressors and mitochondrial toxins in other cell types, it is likely that downregulation of Nrf2-regulated antioxidant and/or phase II conjugating enzymes significantly contributes to increased vulnerability of CRI-G1-RS cells to mitochondrial and oxidative stress, although additional experiments will be required to test this hypothesis. Many questions remain, however. Which Nrf2 target genes are primarily responsible for the observed differences in oxidant and mitochondrial toxin vulnerability? Do Nrf2-independent antioxidant genes, such as Aldh2, contribute to the observed phenotype? What mechanisms mediate upregulation of Nrf2-dependent molecular chaperons in the setting of a global downregulation of Nrf2 pathway? Perhaps most importantly, is there a causal relationship between different changes we observed and – if there is – what is the direction of causality? Further work will be required to answer these questions. However, the gene expression changes we uncovered raise several interesting possibilities.

First, downregulation of Nrf2 pathway in RS cells may be secondary to the lower oxidative stress these cells show at baseline (Fig. 4A); this possibility is supported by the fact that, despite baseline differences in Nrf2 activity, induced Nrf2 response did not significantly differ between the two cell types (Fig. 7). Basal activation of Nrf2 pathway in RR cells would thus represent a hormetic response, where a low dose of a toxic agent results in induction of a beneficial response that protects against a larger dose of the same agent [47]. It is not clear, however, what molecular changes in RR cells underlie the pro-oxidant baseline state observed in this cell type. Second, it is possible that downregulation of Nrf2 pathway and consequent decrease in antioxidant enzyme expression are part of the β cell differentiation program; this hypothesis is consistent with the long-established but unexplained fact that mature β cells express antioxidant enzymes at a much lower level than other cell types. Interestingly, it was recently reported that suppression of K_ATP_ channel activity protects murine pancreatic β cells against oxidative stress through upregulation of antioxidant enzymes superoxide dismutase, glutathione peroxidase and catalase [48], but the mechanism underlying these antioxidant enzyme expression changes was not identified. In our microarray dataset, there was a small but statistically significant upregulation of *Kcnj11* (pore-forming Kir6.2 subunit of the β cell K_ATP_ channel) in oxidant-vulnerable CRI-G1-RS cells, which was associated with increased K_ATP_ current density in this cell type (unpublished preliminary data). The data presented here are thus consistent with a model, which we are currently testing, that an increase in the plasma membrane K_ATP_ current (which is associated with β cell differentiation [42]) inhibits Nrf2 pathway activity and downregulates antioxidant enzyme expression, resulting in a greater vulnerability to metabolic and oxidative stress. If true, this mechanism would have implications beyond β cells and diabetes; for example, deletion of Kir6.2 protects substantia nigra dopaminergic neurons against cell death induced by chronic treatment with MPTP (Parkinsonian neurotoxin and mitochondrial complex I inhibitor [49]), but the molecular mechanism underlying this phenomenon is not well understood. Third, downregulation of Nrf2 pathway may be downstream of integrin/FAK signaling. Flat, surface-adherent morphology of oxidant-susceptible CRI-G1-RS cells (Fig. 1B) is associated with gene expression changes in integrin/FAK signaling pathways (Supplemental Figs. S8 and S9). Integrin clustering and recruitment of FAK to focal adhesions result in activation of multiple downstream signaling cascades including pro-survival PI3K/Akt pathway, which can activate Nrf2 signaling [50], [51]; thus, increased cell adherence would be predicted to result in upregulation, not downregulation, of Nrf2 pathway. However, we observed no change in levels of total Akt or phosphorylated (active) Akt between CRI-G1-RR and CRI-G1-RS cells (Supplemental Fig. S10). Further work will be required to establish whether integrin/FAK signaling is indeed increased or decreased in CRI-G1-RS cells and whether it plays an important role in the regulation of Nrf2 pathway.

What is the physiologic and pathophysiologic role of Nrf2 signaling in β cells? Oxidative stress is thought to play a significant role in the pathogenesis of both type 1 and type 2 diabetes; thus, the low expression level of antioxidant enzymes in β cells – possibly reflecting the low basal activity of Nrf2 pathway, as in CRI-G1-RS cell line – renders these cells vulnerable to the oxidant-induced cell death. Indeed, upregulation of endogenous antioxidant enzymes protects β cells against H_2_O_2_-induced cell death *in vitro* and streptozotocin-induced β cell destruction *in vivo* [48], and was thus proposed as a promising strategy for treatment and/or prevention of diabetes. However, ROS derived from glucose metabolism also act as a signal in GSIS [9]; by blunting glucose-mediated ROS production, upregulation of endogenous antioxidant defenses might compromise β cell function [43]. For example, overexpression of H_2_O_2_-scavenging enzymes catalase and glutathione peroxidase actually sensitizes mice to insulin resistance and diabetes [52], [53]. In this context, it is interesting that downregulation of endogenous antioxidant defenses in CRI-G1 cells was accompanied by increased expression of markers of β cell differentiation (Fig. 9) and reconstitution of GSIS (Fig. 10). It will thus be important to directly examine the effect of Nrf2 pathway activation on GSIS and other aspects of β cell function. More generally, the role of ROS in cellular signaling may limit the physiologic range of antioxidant enzyme expression levels in neuroendocrine cells, thus rendering them particularly vulnerable to oxidative stress. Consistent with this idea, glutathione concentration in neurons is low [54], [55] and activation of Nrf2 pathway in the brain, while neuroprotective, primarily occurs in astrocytes [56]. Importantly, ARE/Keap1/Nrf2 pathway regulates the expression of a large battery of antioxidant and phase I/II conjugation enzymes. By elucidating the effect of individual Nrf2 target genes on (1) β cell and/or neuronal signaling and (2) cell survival, it may be possible to identify individual genes or pathways that decrease oxidative stress vulnerability without interfering with cell function and thus constitute better therapeutic targets than the Nrf2 pathway as a whole.

In summary, we have isolated a novel CRI-G1-RS subclone of the CRI-G1 insulinoma cell line that shows increased expression of β cell differentiation markers, reconstitution of GSIS, decreased activity of ARE/Keap1/Nrf2 pathway, and increased vulnerability to mitochondrial dysfunction and oxidative stress compared to the parental CRI-G1-RR cells. CRI-G1-RR and -RS cell lines thus provide an excellent experimental model for testing of the recently proposed hypothesis that activation of Nrf2 pathway impairs glucose sensing and insulin release by β cells [43]. In addition, the availability of these cell lines will facilitate identification of individual genes and pathways that modulate vulnerability to oxidative stress and thus represent potential therapeutic targets for diabetes and neurodegenerative diseases.

## Materials and Methods

### Cell culture

CRI-G1 cell line was obtained from European Collection of Cell Cultures and maintained in DMEM with GlutaMAX, 4.5 g/L glucose, and 110 mg/L sodium pyruvate (Invitrogen) supplemented with 10% heat inactivated FBS (Omega Scientific) and 10 µg/mL penicillin/10 units/mL streptomycin (UCSF Cell Culture Facility). Cells were initially passaged once a week, allowing cultures to reach relatively high densities; those culturing conditions foster changes in cell morphology and other properties (see Results for additional details). After isolation of a novel CRI-G1-RS subclone, both the original clone (renamed CRI-G1-RR for clarity) and the novel subclone were grown at lower densities and passaged twice a week. Under these conditions, the phenotype of each cell line remained stable for >20 passages.

For imaging, cells were grown on glass coverslips (Bellco) coated with poly-L-lysine (Sigma). Images were taken with a Retiga CCD camera on an Olympus 1×71 inverted DIC microscope using QCapture 2.90.1 software (Quantitative Imaging Corp) and were edited with Adobe Photoshop CS3 Version 10.0.1.

### Drugs

Except for staurosporine and oligomycin, which were purchased from Calbiochem, all drugs were obtained from Sigma-Aldrich. Drugs were generally purchased in dry (powder) form, with stock solutions prepared and stored in small aliquots at −20°C; the exceptions were staurosporine (which was purchased as 1 mM stock solution prepared in dymethylsulfoxide [DMSO]) and xanthine oxidase (which was either dissolved fresh before each experiment or prepared as a stock solution stored at −80°C). Stock solutions were as follows: 10 mM rotenone (in DMSO); 20 mM antimycin (in DMSO); 10 mM oligomycin (in DMSO); 20 mM FCCP (carbonyl cyanide-p-trifluoromethoxyphenylhydrazone; in DMSO); 10 mM hypoxanthine (in water; heating to ∼50°C was required for solubilization and re-solubilization after thawing); 1 kU/L xanthine oxidase (in 50 mM K_2_HPO_4_, pH = 7.25); 2.5 mM L-sulforaphane (in DMSO); and 10 mM *tert*-butylhydroquinone (tBHQ; in DMSO). Antimycin and oligomycin are sold as mixtures of different isoforms, each with a unique molecular weight. Molar concentration was thus calculated based on the molecular weight of a dominant isoform (antimycin A and oligomycin A respectively); the corresponding stock solution mass concentrations were 11.0 mg/mL for antimycin and 7.9 mg/mL for oligomycin.

### Viability and cytotoxicity assays

For cell viability/toxicity assays, cells were grown in a Phenol Red-free media that was otherwise identical in composition to the regular maintenance media. Cells were seeded on 96-well clear (for colorimetric assay) or white (for luminescence assays) tissue culture plates (1–2×10^4^ cells/well, 4 wells/condition) and treated with drugs 18–24 h later. Drug solutions were prepared from stock solutions and applied either at the final concentration (100 µL/well) following aspiration of original plating media, or as a 20 µL volume of a 5× solution (prepared in media) without aspiration of original plating media (for final volume of 100 µL). Viability and toxicity assays were typically performed 18–24 hours later, but longer treatments (up to 5 days) were done in some experiments. Cell viability was determined by CellTiter 96 AQ_ueous_ One Solution Cell Proliferation Assay (Promega) according to the manufacturer's instructions. This assay measures the absorbance of a formazan, which is generated by mitochondrial metabolism and is directly proportional to the number of living cells in the well. Cell death was determined by CytoTox-Glo™ Assay (Promega) according to the manufacturer's instructions; this assay measures the activity of a “dead-cell” protease released into the media from membrane-compromised cells. Apoptosis was determined by measuring activity of caspases 3 and 7 with Caspase-Glo™ 3/7 Assay (Promega) according to the manufacturer's instructions, except that cell lysis was performed in 50 µL (instead of 100 µL) of culture media to minimize the reagent use. Data were analyzed with GraphPad Prism statistical software using two-way ANOVA followed by ad-hoc Bonferroni post-test.

### RNA preparation, microarray hybridization and data analysis

Total RNA was isolated using RNeasy Kit (Qiagen) with contaminating genomic DNA removed during the isolation by an on-column DNase digestion step; six independent replicates were prepared from each cell type. cDNA synthesis, RNA amplification, biotin labeling, and aRNA fragmentation were performed using MessageAmpII Biotin Enhanced Single Round aRNA Amplification Kit (Ambion) according to the manufacturer's instructions. The quality and/or size distribution of total RNA, aRNA, and fragmented aRNA were evaluated by microfluidic gel electrophoresis with a bioanalyzer (Agilent) prior to hybridization. Labeled cRNA samples were hybridized to Affymetrix Rat 230 2.0 arrays, stained, and scanned according to the manufacturer's instructions.

All analyses were performed using the freely available R language [57]. Array quality was analyzed using the affyPLM package [58]. The data were normalized by a robust multi-chip averaging method [59]. Limma package [60], [61] in Bioconductor [62] was used to fit a linear model with log_2_ expression as response variable and cell type as the independent variable. P-values were adjusted by controlling the false discovery rate [63]. A change in gene expression was identified as significant if the false discovery rate was less than 0.05, meaning that fewer than 5% of false findings would be expected among the genes declared to be differentially expressed. The raw image and processed expression values for our dataset are posted in a MIAME-compliant format at NCBI's Gene Expression Omnibus database (accession number GSE19948).

GOstat [23] was used to search for significant GO terms for the significant genes with at least two fold change in expression. The data were also analyzed using Ingenuity Pathways Analysis (Ingenuity® Systems, www.ingenuity.com). The Functional Analysis identified the biological functions that were most significant to the data set. Molecules from the dataset that met the p-value cutoff of 0.001 and were associated with biological functions in Ingenuity's Knowledge Base were considered for the analysis. Right-tailed Fisher's exact test was used to calculate a p-value determining the probability that each biological function and/or disease assigned to that data set is due to chance alone. Canonical pathways analysis identified the pathways from the Ingenuity Pathways Analysis library of canonical pathways that were most significant to the data set. Molecules from the data set that met the p-value cutoff of 0.001 and were associated with a canonical pathway in Ingenuity's Knowledge Base were considered for the analysis. The significance of the association between the data set and the canonical pathway was measured in 2 ways: 1) A ratio of the number of molecules from the data set that map to the pathway divided by the total number of molecules that map to the canonical pathway; 2) Fisher's exact test was used to calculate a p-value determining the probability that the association between the genes in the dataset and the canonical pathway is explained by chance alone. Pathways are graphical representations of the molecular relationships between molecules. Molecules are represented as nodes, and the biological relationship between two nodes is represented as an edge (line). All edges are supported by at least 1 reference from the literature, from a textbook, or from canonical information stored in the Ingenuity Pathways Knowledge Base. Human, mouse, and rat orthologs of a gene are stored as separate objects in the Ingenuity Pathways Knowledge Base, but are represented as a single node in the network. The intensity of the node color indicates the degree of up- (red) or down- (green) regulation. Nodes are displayed using various shapes that represent the functional class of the gene product.

### qRT-PCR

Five independent biological samples of each cell type were used for qRT-PCR analyses. Total RNA was isolated using RNeasy Protect Mini Kit (Qiagen), with RNA integrity (28S/18S) confirmed by running an aliquot of each sample on a 1% agarose mini-gel. mRNA (1 µg/sample) was reverse transcribed to cDNA using Taqman Reverse Transcription Reagents kit (Applied Biosystems). qPCR reactions were carried out in an Opticon DNA Engine 2 Continuous Fluorescence Detection System (Biorad) using 10 ng sample in a 25 µL, 98-well format. TaqMan universal PCR master mix and predesigned TaqMan PCR primer and probe sets were purchased from Applied Biosystems for all genes tested (Supplemental Table S2). Rat ACTB (actin, beta) Endogenous Control FAM Dye/MGB Probe (Applied Biosystems) was used for normalization. The common reference cDNA, used to prepare the standard curve for all reactions, was generated by mixing an equal amount of cDNA obtained from independent biological replicates of each cell type. Three technical replicates for each sample were run on one plate and 2 replicate plates were performed for each gene; thus, a total of 6 technical replicates were performed for each sample and each gene. For analysis, all 6 values were averaged, with mean used for subsequent statistical analysis performed with GraphPad Prism statistical software using two-tailed Student's t-tests.

### Western blotting

Whole cell lysates were prepared by incubating resuspended cell pellets in RIPA lysis buffer (0.5% sodium deoxycholate, 0.1% sodium dodecylsulfate, 1% Triton X-100, 150 mM NaCl, 50 mM TrisCl, 1 mM EDTA; pH = 7.4) supplemented with Complete™ Protease Inhibitor Cocktail (Roche) for 15 min on ice; crude lysates were centrifuged (15,000 g for 10 min at 4°C) to remove insoluble/particulate matter. Nuclear and cytoplasmic fractions were prepared using the NE-PER kit (Pierce) according to the manufacturer's instructions. The protein concentration of each sample was assayed relative to the bovine serum albumin (BSA) standard with the CBB Assay kit (Dojindo); approximately 25 µg of total protein was eventually loaded in each lane. After solubilization with LDS sample buffer (Invitrogen) supplemented with TCEP reducing reagent (Pierce; final concentration 12 mM), samples were heated for 10 min at 70°C, electrophoretically resolved with 10% or 4–12% (for Nrf2) NuPAGE precast gels (Invitrogen), and electroblotted to nitrocellulose membranes (0.2 A for 5 hours at 4°C). Three percent nonfat dried milk in TBS (150 mM NaCl, 20 mM Tris Cl; pH = 7.4) was used for blocking, washing, and dilution of primary and secondary antibodies, except for DJ-1, Akt and P-Akt where primary antibody incubation was performed in TBS supplemented with 5% w/v BSA and 0.1% Tween. Membranes were blocked for 1 h at room temperature (RT); incubated with primary antibodies for 2 h at RT (Keap1, COXIV, cytochrome c, tubulin, tom20 and lamin A), 2–4 h at RT (Nrf2), or overnight at 4°C (DJ-1, Akt and P-Akt); washed for 1 h at RT; incubated with corresponding secondary antibody for 1 h at RT; and washed for 45 min at RT. Following a final wash in TBS with 0.1% Tween for 15 min at RT and a water rinse, protein-antibody complexes were detected using an ECL chemiluminescent kit (Pierce Biotechnology) and CL-XPosure Film (Thermo Scientific) with a Konica SRX-101A developer.

The following primary antibodies were used: rabbit polyclonal anti-PARK7/DJ-1, 1 µg/mL (GenWay Biotech); mouse monoclonal anti-Keap1, 1 µg/mL (ProteinTech Group); mouse monoclonal anti-COXIV 20E8, 0.1 µg/mL (Abcam); mouse monoclonal anti-cytochrome c, 0.5 µg/mL (BD Pharmingen); rabbit polyclonal anti-Tom20 FL-145, 0.07 µg/mL, and rabbit polyclonal Nrf2 H300, 0.5 µg/mL (Santa Cruz Biotechnology); rabbit polyclonal anti-Aldh2, 1∶10000 (gift of Dr. Henry Weiner, Purdue University); rabbit polyclonal anti-Akt and anti-phospho-Akt, 1∶1000 (Cell Signaling); mouse monoclonal anti-tubulin clone DM1A (ascites fluid), 1 µg/mL, and rabbit polyclonal anti-lamin A C-terminal, 1 µg/mL (Sigma). Horseradish peroxidase-conjugated anti-mouse and anti-rabbit secondary H+L IgG antibodies were purchased from Jackson ImmunoResearch; anti-mouse antibody was used at 0.8 µg/mL, while anti-rabbit antibody was used at either 8 µg/mL (for anti-PARK7/DJ-1 and anti-Nrf2) or 0.8 µg/mL (for anti-Tom20, anti-Aldh2, anti-Akt, anti-P-Akt. and anti-lamin A).

ImageJ software (http://rsb.info.nih.gov/ij/) was used for band quantification by densitometry. Data were analyzed with GraphPad Prism statistical software using two-tailed t-test (for comparison of baseline protein levels) or two-way ANOVA with ad hoc Bonferroni post-test (for comparison of drug treatment effects between the two cell types).

### 4-HNE immunostaining

Cells were grown overnight on poly-L-lysine-coated glass coverslips, incubated with hypoxanthine (0.5 mM)/xanthine oxidase (16 U/L) for 1 hour, washed once with PBS, and fixed in 4% formaldehyde 4% sucrose in PBS for 15 minutes at room temperature; coverslips were stored in PBS at 4°C until immunostaining. Cells were blocked in PBS with 0.1% Triton-X and 5% normal goat serum for 1 hour at room temperature, incubated in blocking solution with 4 µg/mL rabbit anti-4-HNE (Alpha Diagnostic) at 4°C overnight, washed 3 times in PBS with 0.1% Triton-X, and incubated in blocking solution with 2 µg/mL Alexa Fluor 488-conjugated goat anti-rabbit IgG (H+L; Invitrogen) for 2 hours at room temperature. Coverslips were mounted on glass slides with Vectashield mounting medium containing DAPI nuclear counter stain (Vector Laboratories). Images were taken with Zeiss LSM 510 imaging software (version 4.2) using 20× and 63× objectives on Zeiss LSM 510 NLO confocal microscope; they were edited with Adobe Photoshop CS3 Version 10.0.1.

### Glutathione measurement

The concentration of reduced (GSH) and total (GSH+GSSG) glutathione was determined using the GSH-Glo™ Glutathione Assay kit (Promega) according to the manufacturer's instructions; this luminescence-based assay is based on the conversion of a luciferin derivative into luciferin, catalyzed by glutathione S-transferase in the presence of glutathione. Frozen cell pellets were resuspended in 200 µL ice-cold phosphate-buffered saline supplemented with Complete™ Protease Inhibitor Cocktail (Roche). 10 µL aliquot of each cell suspension (3 technical replicates/sample) was used to measure GSH. Total glutathione was measured by adding reducing agent TCEP (Pierce; 1 mM final concentration) to 3 separate aliquots of each suspension to convert GSSG to GSH prior to GSH measurement; the amount of TCEP added did not significantly alter the total volume. Readings were then normalized to protein concentration measured by CBB kit (Dojindo) in a lysate prepared from an aliquot of each cell suspension (as described for whole cell lysate in the Western blotting section). The amount of GSSG in the sample was calculated by halving the difference between the total and reduced glutathione; two RS cell samples that had negative/undetectable GSSG levels were excluded from the GSH/GSSG ratio analysis. Data were analyzed with GraphPad Prism statistical software using two-way ANOVA followed by ad-hoc Bonferroni post-tests.

### Insulin ELISA

Cells were plated in a 12-well dish (5×10^4^ cells/well). The next day, cells were washed twice with KRBB 2% BSA (Krebs-Ringer Bicarbonate Buffer – 130 mM NaCl, 5 mM KCl, 1.25 mM KH_2_PO_4_, 1.25 mM MgSO_4_, 2.68 mM CaCl_2_, 5.26 mM NaHCO_3_,10 mM HEPES; pH = 7.4), incubated in KRBB 2% BSA at 37°C for 3 h, and then incubated in KRBB 2% BSA with or without 20 mM glucose for 30 min; the supernatant from 30 min incubation, which contained the secreted insulin, was collected, spun down to remove debris, and stored in aliquots at −80°C. To measure residual intracellular insulin, cells that remained in the dish were scraped, pelleted, and lysed in 200 µL acid ethanol (0.2 M HCl in 75% ethanol) overnight at 4°C. The lysate was spun down to remove debris, neutralized with 0.2 M NaOH, aliquoted, and stored at −80°C. Insulin was measured using the Rat/Mouse Insulin ELISA kit (Millipore); values for both secreted and intracellular insulin were normalized to the total DNA content (measured by DNA spectrometry) in each lysate. Data were analyzed with GraphPad Prism statistical software using two-way ANOVA followed by ad-hoc Bonferroni post-tests.

## Supporting Information

Figure S1 (0.13 MB PPT)

Figure S2 (0.25 MB PPT)

Figure S3 (0.10 MB PPT)

Figure S4 (2.07 MB PDF)

Figure S5 (0.83 MB PPT)

Figure S6 (0.09 MB PPT)

Figure S7 (2.13 MB PDF)

Figure S8 (3.27 MB PPT)

Figure S9 (2.41 MB PPT)

Figure S10 (0.08 MB PPT)

Table S1 (0.04 MB XLS)

Table S2 (0.03 MB XLS)

## References

[1] Wallace DC (2005). A mitochondrial paradigm of metabolic and degenerative diseases,
aging, and cancer: a dawn for evolutionary medicine.. Annu Rev Genet.

[2] Petersen KF, Befroy D, Dufour S, Dziura J, Ariyan C (2003). Mitochondrial dysfunction in the elderly: possible role in
insulin resistance.. Science.

[3] Petersen KF, Dufour S, Befroy D, Garcia R, Shulman GI (2004). Impaired mitochondrial activity in the insulin-resistant
offspring of patients with type 2 diabetes.. N Engl J Med.

[4] Houstis N, Rosen ED, Lander ES (2006). Reactive oxygen species have a causal role in multiple forms of
insulin resistance.. Nature.

[5] Soejima A, Inoue K, Takai D, Kaneko M, Ishihara H (1996). Mitochondrial DNA is required for regulation of
glucose-stimulated insulin secretion in a mouse pancreatic beta cell line,
MIN6.. J Biol Chem.

[6] Brissova M, Shiota M, Nicholson WE, Gannon M, Knobel SM (2002). Reduction in pancreatic transcription factor PDX-1 impairs
glucose-stimulated insulin secretion.. J Biol Chem.

[7] Gauthier BR, Brun T, Sarret EJ, Ishihara H, Schaad O (2004). Oligonucleotide microarray analysis reveals PDX1 as an essential
regulator of mitochondrial metabolism in rat islets.. J Biol Chem.

[8] Leloup C, Magnan C, Benani A, Bonnet E, Alquier T (2006). Mitochondrial reactive oxygen species are required for
hypothalamic glucose sensing.. Diabetes.

[9] Pi J, Bai Y, Zhang Q, Wong V, Floering LM (2007). Reactive oxygen species as a signal in glucose-stimulated insulin
secretion.. Diabetes.

[10] Ristow M (2004). Neurodegenerative disorders associated with diabetes
mellitus.. J Mol Med.

[11] Kopf D, Frolich L (2009). Risk of incident Alzheimer's disease in diabetic patients: a
systematic review of prospective trials.. J Alzheimers Dis.

[12] Xu WL, von Strauss E, Qiu CX, Winblad B, Fratiglioni L (2009). Uncontrolled diabetes increases the risk of Alzheimer's
disease: a population-based cohort study.. Diabetologia.

[13] Ronnemaa E, Zethelius B, Sundelof J, Sundstrom J, Degerman-Gunnarsson M (2009). Glucose metabolism and the risk of Alzheimer's disease and
dementia: a population-based 12 year follow-up study in 71-year-old
men.. Diabetologia.

[14] Hu G, Jousilahti P, Bidel S, Antikainen R, Tuomilehto J (2007). Type 2 diabetes and the risk of Parkinson's
disease.. Diabetes Care.

[15] D'Amelio M, Ragonese P, Callari G, Di Benedetto N, Palmeri B (2009). Diabetes preceding Parkinson's disease onset. A case-control
study.. Parkinsonism Relat Disord.

[16] Carrington CA, Rubery ED, Pearson EC, Hales CN (1986). Five new insulin-producing cell lines with differing secretory
properties.. J Endocrinol.

[17] Hoglinger GU, Carrard G, Michel PP, Medja F, Lombes A (2003). Dysfunction of mitochondrial complex I and the proteasome:
interactions between two biochemical deficits in a cellular model of
Parkinson's disease.. J Neurochem.

[18] Moon Y, Lee KH, Park JH, Geum D, Kim K (2005). Mitochondrial membrane depolarization and the selective death of
dopaminergic neurons by rotenone: protective effect of coenzyme
Q10.. J Neurochem.

[19] Fonck C, Baudry M (2003). Rapid reduction of ATP synthesis and lack of free radical
formation by MPP^+^ in rat brain synaptosomes and
mitochondria.. Brain Res.

[20] Moriscot C, Candel S, Sauret V, Kerr-Conte J, Richard MJ (2007). MnTMPyP, a metalloporphyrin-based superoxide dismutase/catalase
mimetic, protects INS-1 cells and human pancreatic islets from an in vitro
oxidative challenge.. Diabetes Metab.

[21] Brennan AM, Suh SW, Won SJ, Narasimhan P, Kauppinen TM (2009). NADPH oxidase is the primary source of superoxide induced by NMDA
receptor activation.. Nat Neurosci.

[22] Sies H, Akerboom TP (1984). Glutathione disulfide (GSSG) efflux from cells and
tissues.. Methods Enzymol.

[23] Beissbarth T, Speed TP (2004). GOstat: find statistically overrepresented Gene Ontologies within
a group of genes.. Bioinformatics.

[24] Kensler TW, Wakabayashi N, Biswal S (2007). Cell survival responses to environmental stresses via the
Keap1-Nrf2-ARE pathway.. Annu Rev Pharmacol Toxicol.

[25] Nguyen T, Yang CS, Pickett CB (2004). The pathways and molecular mechanisms regulating Nrf2 activation
in response to chemical stress.. Free Radic Biol Med.

[26] Clements CM, McNally RS, Conti BJ, Mak TW, Ting JP (2006). DJ-1, a cancer- and Parkinson's disease-associated protein,
stabilizes the antioxidant transcriptional master regulator
Nrf2.. Proc Natl Acad Sci U S A.

[27] Malhotra D, Thimmulappa R, Navas-Acien A, Sandford A, Elliott M (2008). Decline in NRF2-regulated antioxidants in chronic obstructive
pulmonary disease lungs due to loss of its positive regulator,
DJ-1.. Am J Respir Crit Care Med.

[28] Gan L, Johnson DA, Johnson JA (2010). Keap1-Nrf2 activation in the presence and absence of
DJ-1.. Eur J Neurosci.

[29] Andres-Mateos E, Perier C, Zhang L, Blanchard-Fillion B, Greco TM (2007). DJ-1 gene deletion reveals that DJ-1 is an atypical
peroxiredoxin-like peroxidase.. Proc Natl Acad Sci U S A.

[30] Shendelman S, Jonason A, Martinat C, Leete T, Abeliovich A (2004). DJ-1 is a redox-dependent molecular chaperone that inhibits
alpha-synuclein aggregate formation.. PLoS Biol.

[31] Bonifati V, Rizzu P, van Baren MJ, Schaap O, Breedveld GJ (2003). Mutations in the DJ-1 gene associated with autosomal recessive
early-onset parkinsonism.. Science.

[32] Inberg A, Linial M (2010). Protection of pancreatic β-cells from various stress
conditions is mediated by DJ-1.. J Biol Chem Epub ahead of print (PMID: 20516060).

[33] Chen CH, Budas GR, Churchill EN, Disatnik MH, Hurley TD (2008). Activation of aldehyde dehydrogenase-2 reduces ischemic damage to
the heart.. Science.

[34] Wang B, Wang J, Zhou S, Tan S, He X (2008). The association of mitochondrial aldehyde dehydrogenase gene
(ALDH2) polymorphism with susceptibility to late-onset Alzheimer's
disease in Chinese.. J Neurol Sci.

[35] Ohsawa I, Nishimaki K, Yasuda C, Kamino K, Ohta S (2003). Deficiency in a mitochondrial aldehyde dehydrogenase increases
vulnerability to oxidative stress in PC12 cells.. J Neurochem.

[36] Ohsawa I, Nishimaki K, Murakami Y, Suzuki Y, Ishikawa M (2008). Age-dependent neurodegeneration accompanying memory loss in
transgenic mice defective in mitochondrial aldehyde dehydrogenase 2
activity.. J Neurosci.

[37] Cho HY, Reddy SP, Debiase A, Yamamoto M, Kleeberger SR (2005). Gene expression profiling of NRF2-mediated protection against
oxidative injury.. Free Radic Biol Med.

[38] Reisman SA, Yeager RL, Yamamoto M, Klaassen CD (2009). Increased Nrf2 activation in livers from Keap1-knockdown mice
increases expression of cytoprotective genes that detoxify electrophiles
more than those that detoxify reactive oxygen species.. Toxicol Sci.

[39] Thimmulappa RK, Mai KH, Srisuma S, Kensler TW, Yamamoto M (2002). Identification of Nrf2-regulated genes induced by the
chemopreventive agent sulforaphane by oligonucleotide
microarray.. Cancer Res.

[40] Hu R, Xu C, Shen G, Jain MR, Khor TO (2006). Gene expression profiles induced by cancer chemopreventive
isothiocyanate sulforaphane in the liver of C57BL/6J mice and C57BL/6J/Nrf2
(−/−) mice.. Cancer Lett.

[41] Cerf ME (2006). Transcription factors regulating β-cell
function.. Eur J Endocrinol.

[42] Miyazaki S, Yamato E, Miyazaki J (2004). Regulated expression of pdx-1 promotes in vitro differentiation
of insulin-producing cells from embryonic stem cells.. Diabetes.

[43] Pi J, Zhang Q, Fu J, Woods CG, Hou Y (2009). ROS signaling, oxidative stress and Nrf2 in pancreatic beta-cell
function.. Toxicol Appl Pharmacol.

[44] Tiedge M, Lortz S, Drinkgern J, Lenzen S (1997). Relation between antioxidant enzyme gene expression and
antioxidative defense status of insulin-producing cells.. Diabetes.

[45] Donath MY, Ehses JA, Maedler K, Schumann DM, Ellingsgaard H (2005). Mechanisms of β-cell death in type 2
diabetes.. Diabetes.

[46] Rolo AP, Palmeira CM (2006). Diabetes and mitochondrial function: role of hyperglycemia and
oxidative stress.. Toxicol Appl Pharmacol.

[47] Mattson MP, Cheng A (2006). Neurohormetic phytochemicals: Low-dose toxins that induce
adaptive neuronal stress responses.. Trends Neurosci.

[48] Gier B, Krippeit-Drews P, Sheiko T, Aguilar-Bryan L, Bryan J (2009). Suppression of K_ATP_ channel activity protects murine
pancreatic β cells against oxidative stress.. J Clin Invest.

[49] Liss B, Haeckel O, Wildmann J, Miki T, Seino S (2005). K-ATP channels promote the differential degeneration of
dopaminergic midbrain neurons.. Nat Neurosci.

[50] Nakaso K, Yano H, Fukuhara Y, Takeshima T, Wada-Isoe K (2003). PI3K is a key molecule in the Nrf2-mediated regulation of
antioxidative proteins by hemin in human neuroblastoma
cells.. FEBS Lett.

[51] Wang L, Chen Y, Sternberg P, Cai J (2008). Essential roles of the PI3 kinase/Akt pathway in regulating
Nrf2-dependent antioxidant functions in the RPE.. Invest Ophthalmol Vis Sci.

[52] Li X, Chen H, Epstein PN (2006). Metallothionein and catalase sensitize to diabetes in nonobese
diabetic mice: reactive oxygen species may have a protective role in
pancreatic β-cells.. Diabetes.

[53] McClung JP, Roneker CA, Mu W, Lisk DJ, Langlais P (2004). Development of insulin resistance and obesity in mice
overexpressing cellular glutathione peroxidase.. Proc Natl Acad Sci U S A.

[54] Makar TK, Nedergaard M, Preuss A, Gelbard AS, Perumal AS (1994). Vitamin E, ascorbate, glutathione, glutathione disulfide, and
enzymes of glutathione metabolism in cultures of chick astrocytes and
neurons: evidence that astrocytes play an important role in antioxidative
processes in the brain.. J Neurochem.

[55] Raps SP, Lai JC, Hertz L, Cooper AJ (1989). Glutathione is present in high concentrations in cultured
astrocytes but not in cultured neurons.. Brain Res.

[56] Kraft AD, Johnson DA, Johnson JA (2004). Nuclear factor E2-related factor 2-dependent antioxidant response
element activation by *tert*-butylhydroquinone and
sulforaphane occurring preferentially in astrocytes conditions neurons
against oxidative insult.. J Neurosci.

[57] R Development Core Team (2005). R: A language and environment for statistical
computing.. http://www.R-project.org.

[58] Bolstad BM (2004). Low level analysis of high-density oligonucleotide array data:
Background, normalization and summarization..

[59] Irizarry RA, Hobbs B, Collin F, Beazer-Barclay YD, Antonellis KJ (2003). Exploration, normalization, and summaries of high density
oligonucleotide array probe level data.. Biostatistics.

[60] Smyth GK, Gentleman R, Carey V, Dudoit S, Irizarry RA, Huber W (2005). Limma: linear models for microarray data.. Bioinformatics and Computational Biology Solutions using R and
Bioconductor.

[61] Smyth GK (2004). Linear models and empirical Bayes methods for assessing
differential expression in microarray experiments.. Stat Appl Genet Mol Biol.

[62] Gentleman RC, Carey VJ, Bates DM, Bolstad B, Dettling M (2004). Bioconductor: open software development for computational biology
and bioinformatics.. Genome Biol.

[63] Benjamini Y, Hochberg Y (1995). Controlling the false recovery rate: A practical and powerful
approach to multiple testing.. J Royal Stat Soc B.

