# Supplementary material for: Molecular Basis for Vulnerability to Mitochondrial and Oxidative Stress in a Neuroendocrine CRI-G1 Cell Line
Source: PLoS One. 2011 Jan 4;6(1):e14485. doi: 10.1371/journal.pone.0014485 (PMC3020905; doi:10.1371/journal.pone.0014485)
Supplement: Figure S1 — (0.13 MB PPT) [file pone.0014485.s001.ppt]

## Slide 1
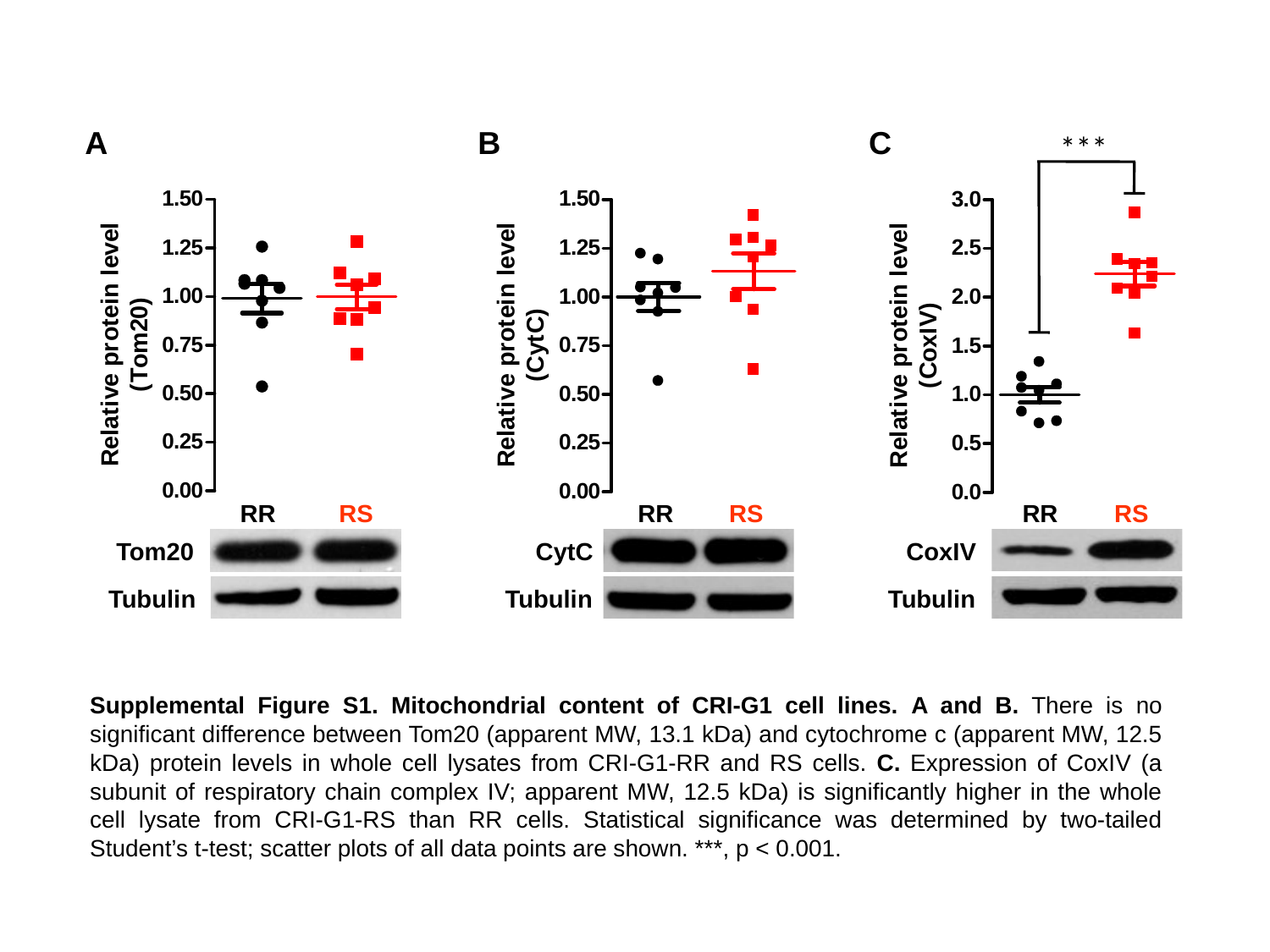

A
B
C
***
RR
RS
CoxIV
Tubulin
RR
RS
Tom20
Tubulin
RR
RS
CytC
Tubulin
# Supplemental Figure S1. Mitochondrial content of CRI-G1 cell lines. A and B. There is no significant difference between Tom20 (apparent MW, 13.1 kDa) and cytochrome c (apparent MW, 12.5 kDa) protein levels in whole cell lysates from CRI-G1-RR and RS cells. C. Expression of CoxIV (a subunit of respiratory chain complex IV; apparent MW, 12.5 kDa) is significantly higher in the whole cell lysate from CRI-G1-RS than RR cells. Statistical significance was determined by two-tailed Student’s t-test; scatter plots of all data points are shown. ***, p < 0.001.
