# Supplementary material for: Molecular Basis for Vulnerability to Mitochondrial and Oxidative Stress in a Neuroendocrine CRI-G1 Cell Line
Source: PLoS One. 2011 Jan 4;6(1):e14485. doi: 10.1371/journal.pone.0014485 (PMC3020905; doi:10.1371/journal.pone.0014485)
Supplement: Figure S2 — (0.25 MB PPT) [file pone.0014485.s002.ppt]

## Slide 1
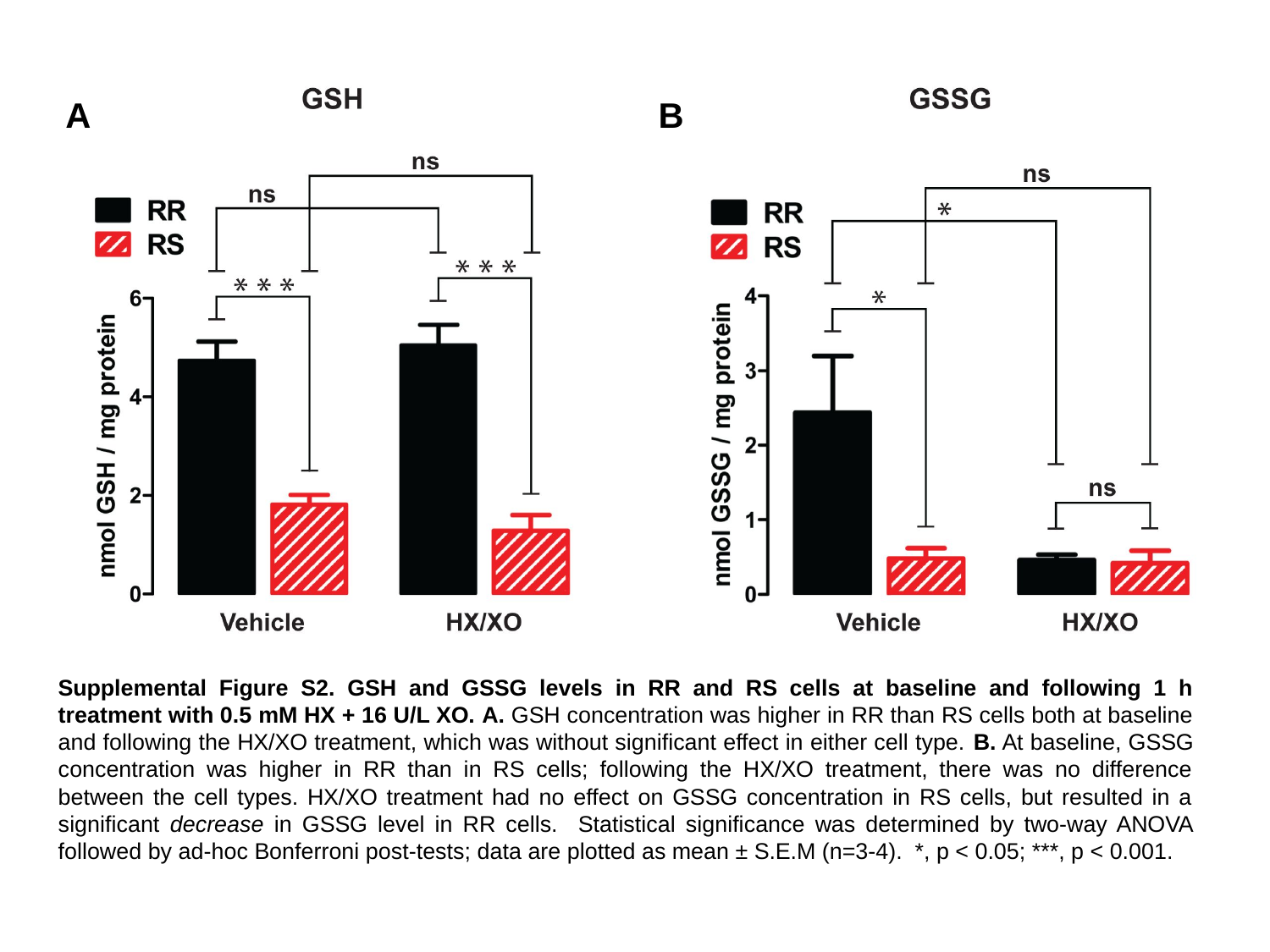

A
B
	Supplemental Figure S2. GSH and GSSG levels in RR and RS cells at baseline and following 1 h treatment with 0.5 mM HX + 16 U/L XO. A. GSH concentration was higher in RR than RS cells both at baseline and following the HX/XO treatment, which was without significant effect in either cell type. B. At baseline, GSSG concentration was higher in RR than in RS cells; following the HX/XO treatment, there was no difference between the cell types. HX/XO treatment had no effect on GSSG concentration in RS cells, but resulted in a significant decrease in GSSG level in RR cells. Statistical significance was determined by two-way ANOVA followed by ad-hoc Bonferroni post-tests; data are plotted as mean ± S.E.M (n=3-4). *, p < 0.05; ***, p < 0.001.
