# Supplementary material for: Molecular Basis for Vulnerability to Mitochondrial and Oxidative Stress in a Neuroendocrine CRI-G1 Cell Line
Source: PLoS One. 2011 Jan 4;6(1):e14485. doi: 10.1371/journal.pone.0014485 (PMC3020905; doi:10.1371/journal.pone.0014485)
Supplement: Figure S3 — (0.10 MB PPT) [file pone.0014485.s003.ppt]

## Slide 1
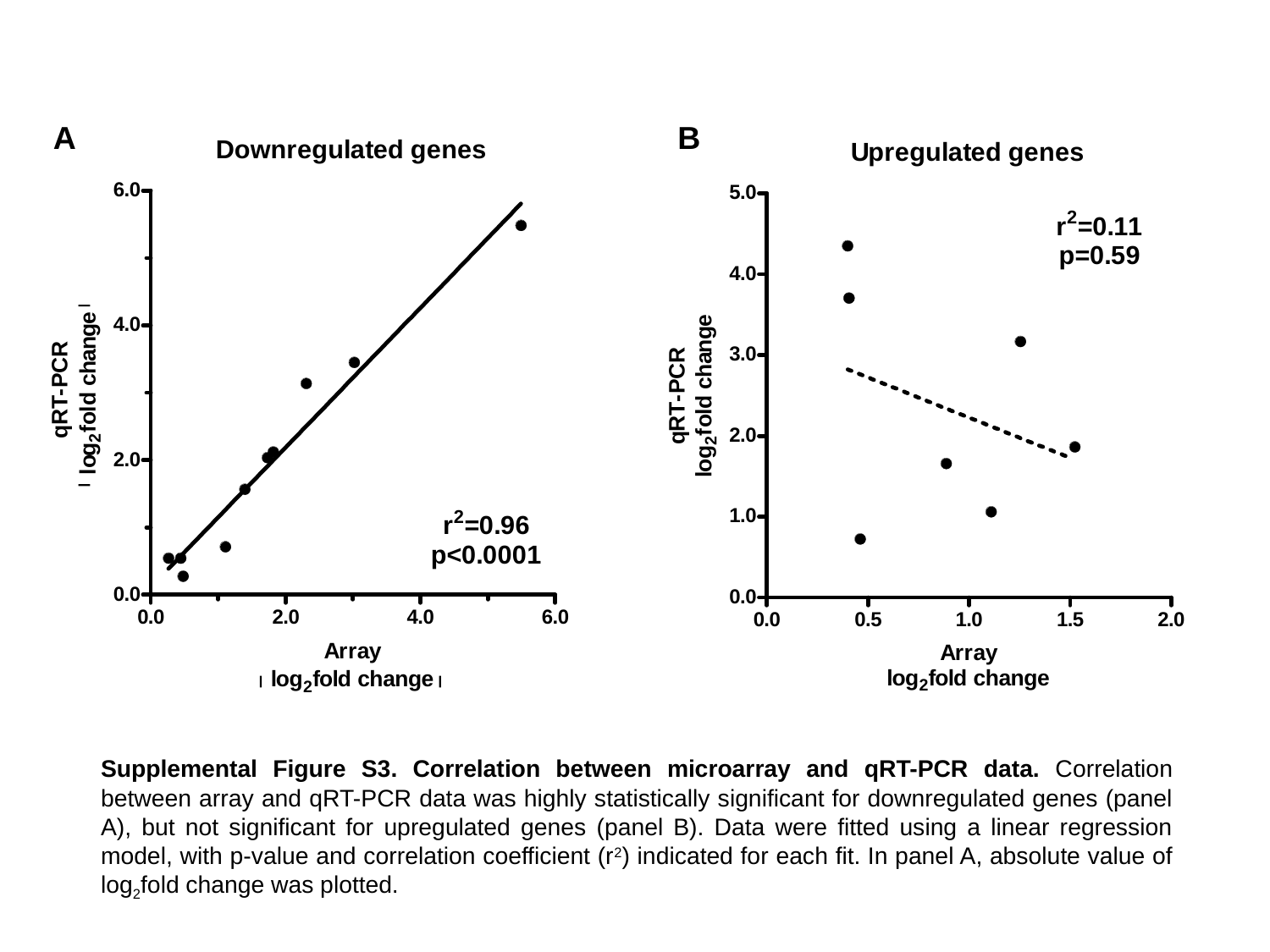

A
B
	Supplemental Figure S3. Correlation between microarray and qRT-PCR data. Correlation between array and qRT-PCR data was highly statistically significant for downregulated genes (panel A), but not significant for upregulated genes (panel B). Data were fitted using a linear regression model, with p-value and correlation coefficient (r2) indicated for each fit. In panel A, absolute value of log2fold change was plotted.
