# Supplementary figures and images for: Molecular Basis for Vulnerability to Mitochondrial and Oxidative Stress in a Neuroendocrine CRI-G1 Cell Line
Source: PLoS One. 2011 Jan 4;6(1):e14485. doi: 10.1371/journal.pone.0014485 (PMC3020905; doi:10.1371/journal.pone.0014485)

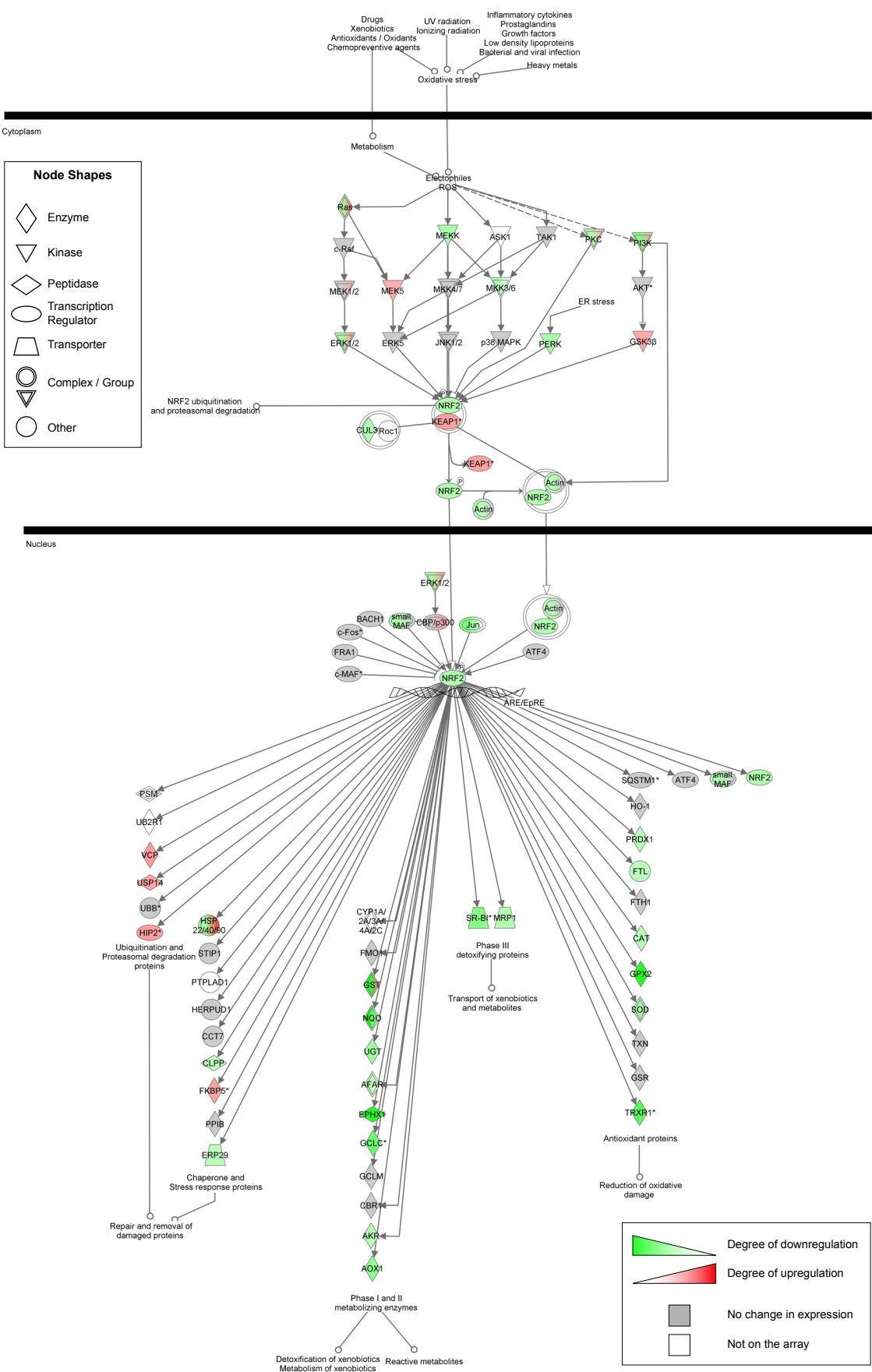

Supplement: Figure S4 — (2.07 MB PDF) [file pone.0014485.s004.pdf]

Extracellular space

Plasma Membrane

Cytoplasm

Nucleus

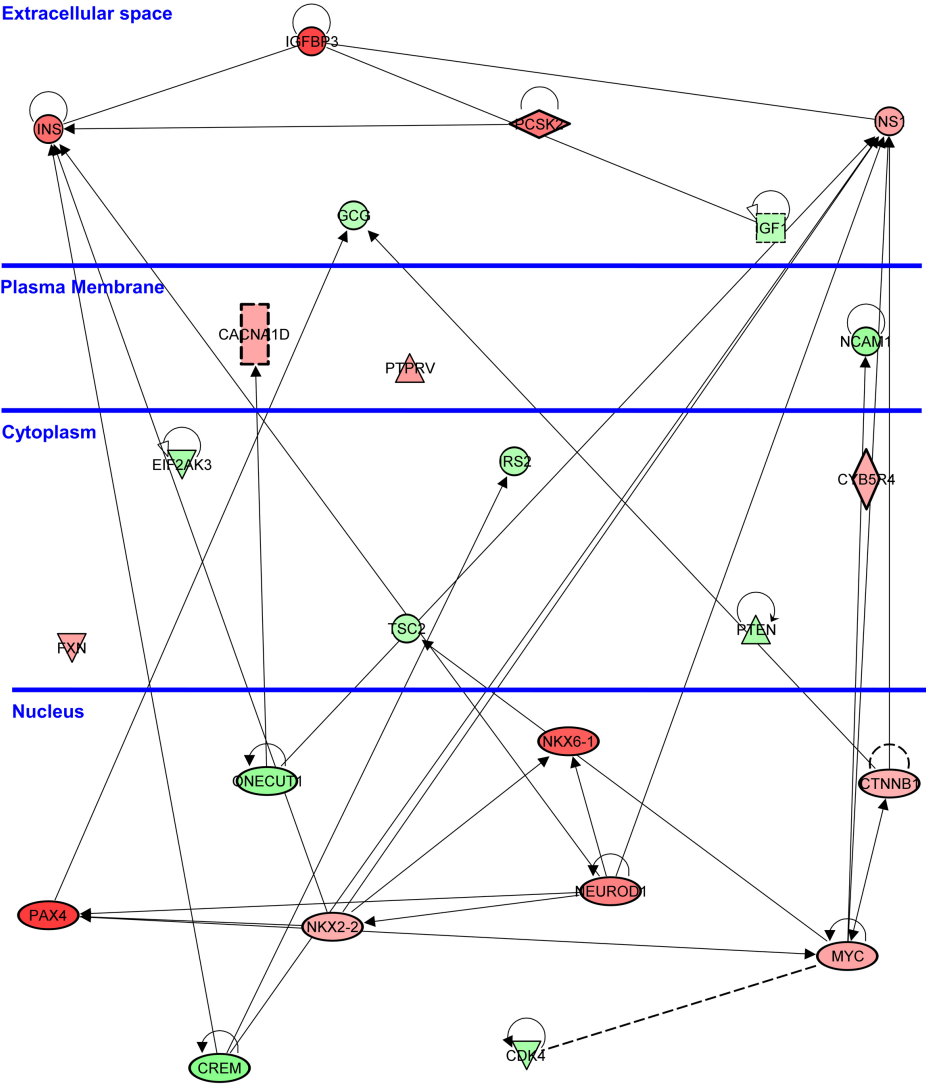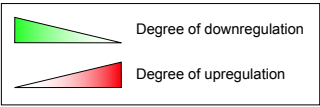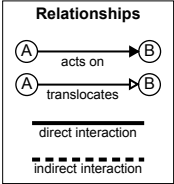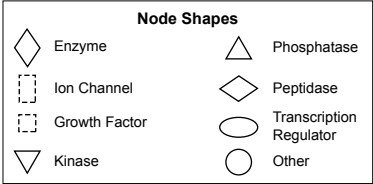

Supplement: Figure S7 — (2.13 MB PDF) [file pone.0014485.s007.pdf]
