# Supplementary material for: Molecular Basis for Vulnerability to Mitochondrial and Oxidative Stress in a Neuroendocrine CRI-G1 Cell Line
Source: PLoS One. 2011 Jan 4;6(1):e14485. doi: 10.1371/journal.pone.0014485 (PMC3020905; doi:10.1371/journal.pone.0014485)
Supplement: Figure S5 — (0.83 MB PPT) [file pone.0014485.s005.ppt]

## Slide 1
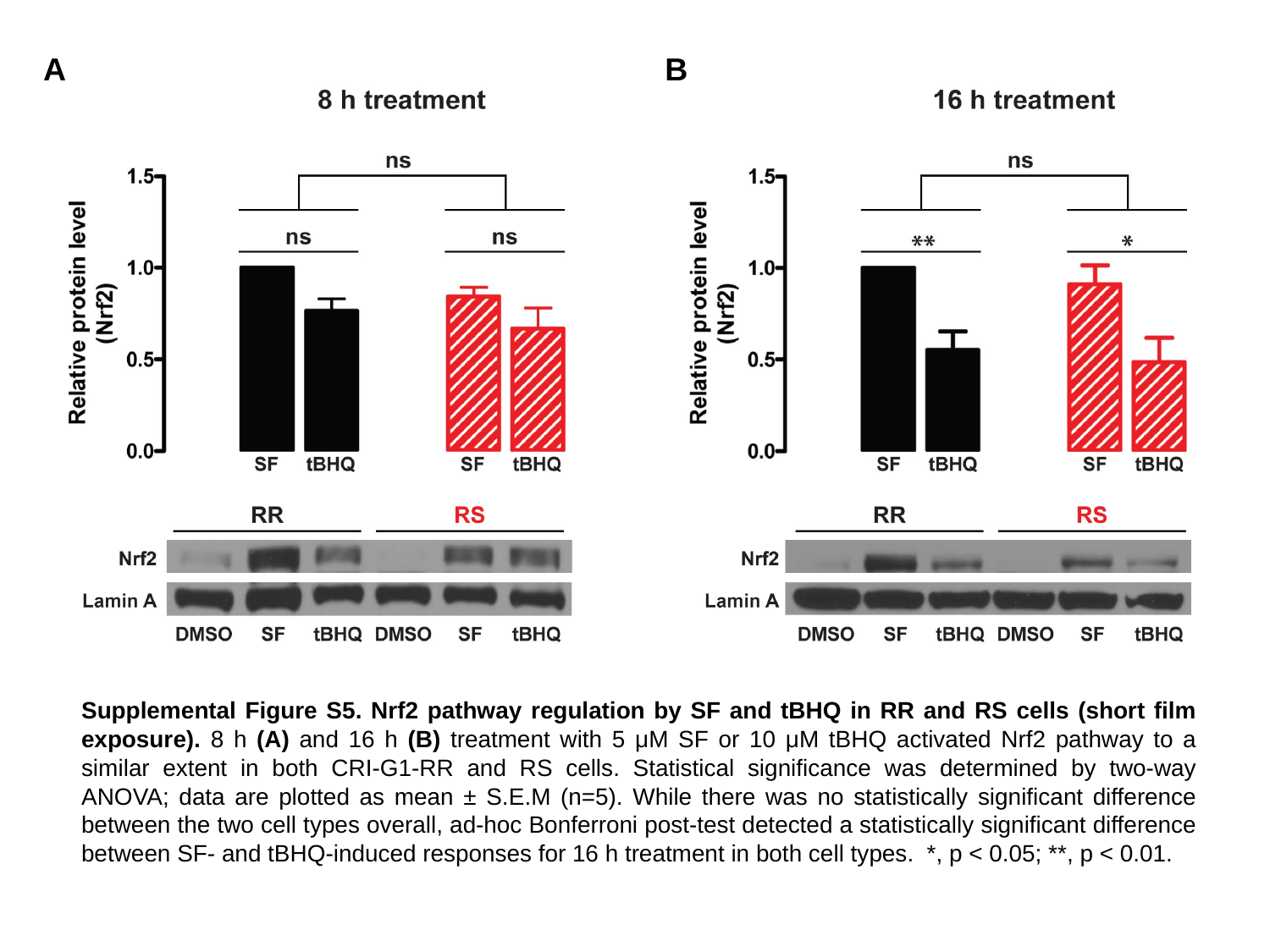

A
B
	Supplemental Figure S5. Nrf2 pathway regulation by SF and tBHQ in RR and RS cells (short film exposure). 8 h (A) and 16 h (B) treatment with 5 μM SF or 10 μM tBHQ activated Nrf2 pathway to a similar extent in both CRI-G1-RR and RS cells. Statistical significance was determined by two-way ANOVA; data are plotted as mean ± S.E.M (n=5). While there was no statistically significant difference between the two cell types overall, ad-hoc Bonferroni post-test detected a statistically significant difference between SF- and tBHQ-induced responses for 16 h treatment in both cell types. *, p < 0.05; **, p < 0.01.
