# Supplementary material for: Molecular Basis for Vulnerability to Mitochondrial and Oxidative Stress in a Neuroendocrine CRI-G1 Cell Line
Source: PLoS One. 2011 Jan 4;6(1):e14485. doi: 10.1371/journal.pone.0014485 (PMC3020905; doi:10.1371/journal.pone.0014485)
Supplement: Figure S6 — (0.09 MB PPT) [file pone.0014485.s006.ppt]

## Slide 1
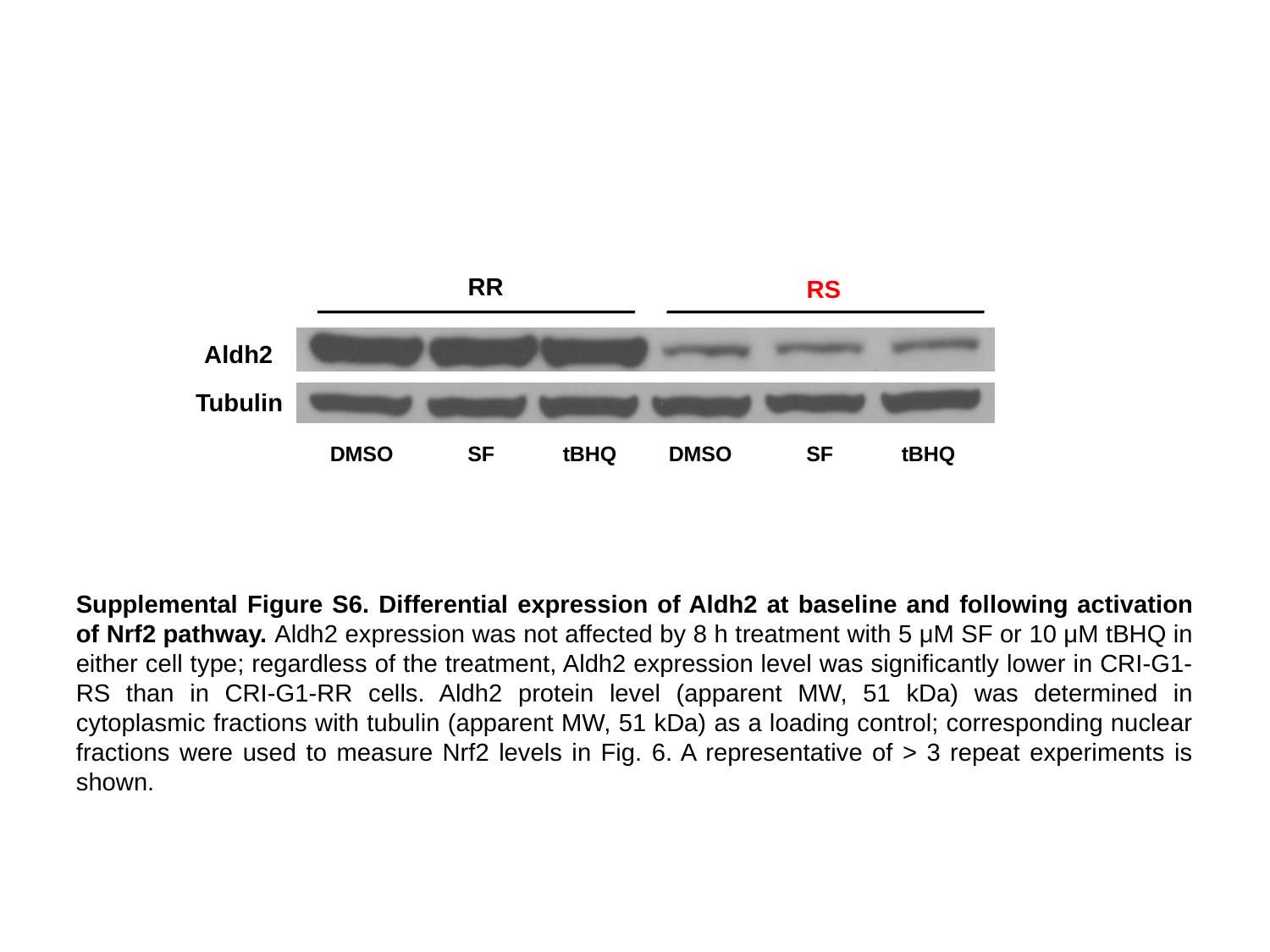

RR
RS
Aldh2
Tubulin
DMSO
SF
tBHQ
DMSO
SF
tBHQ
Supplemental Figure S6. Differential expression of Aldh2 at baseline and following activation of Nrf2 pathway. Aldh2 expression was not affected by 8 h treatment with 5 μM SF or 10 μM tBHQ in either cell type; regardless of the treatment, Aldh2 expression level was significantly lower in CRI-G1-RS than in CRI-G1-RR cells. Aldh2 protein level (apparent MW, 51 kDa) was determined in cytoplasmic fractions with tubulin (apparent MW, 51 kDa) as a loading control; corresponding nuclear fractions were used to measure Nrf2 levels in Fig. 6. A representative of > 3 repeat experiments is shown.
