# Supplementary material for: Molecular Basis for Vulnerability to Mitochondrial and Oxidative Stress in a Neuroendocrine CRI-G1 Cell Line
Source: PLoS One. 2011 Jan 4;6(1):e14485. doi: 10.1371/journal.pone.0014485 (PMC3020905; doi:10.1371/journal.pone.0014485)
Supplement: Figure S9 — (2.41 MB PPT) [file pone.0014485.s009.ppt]

## Slide 1
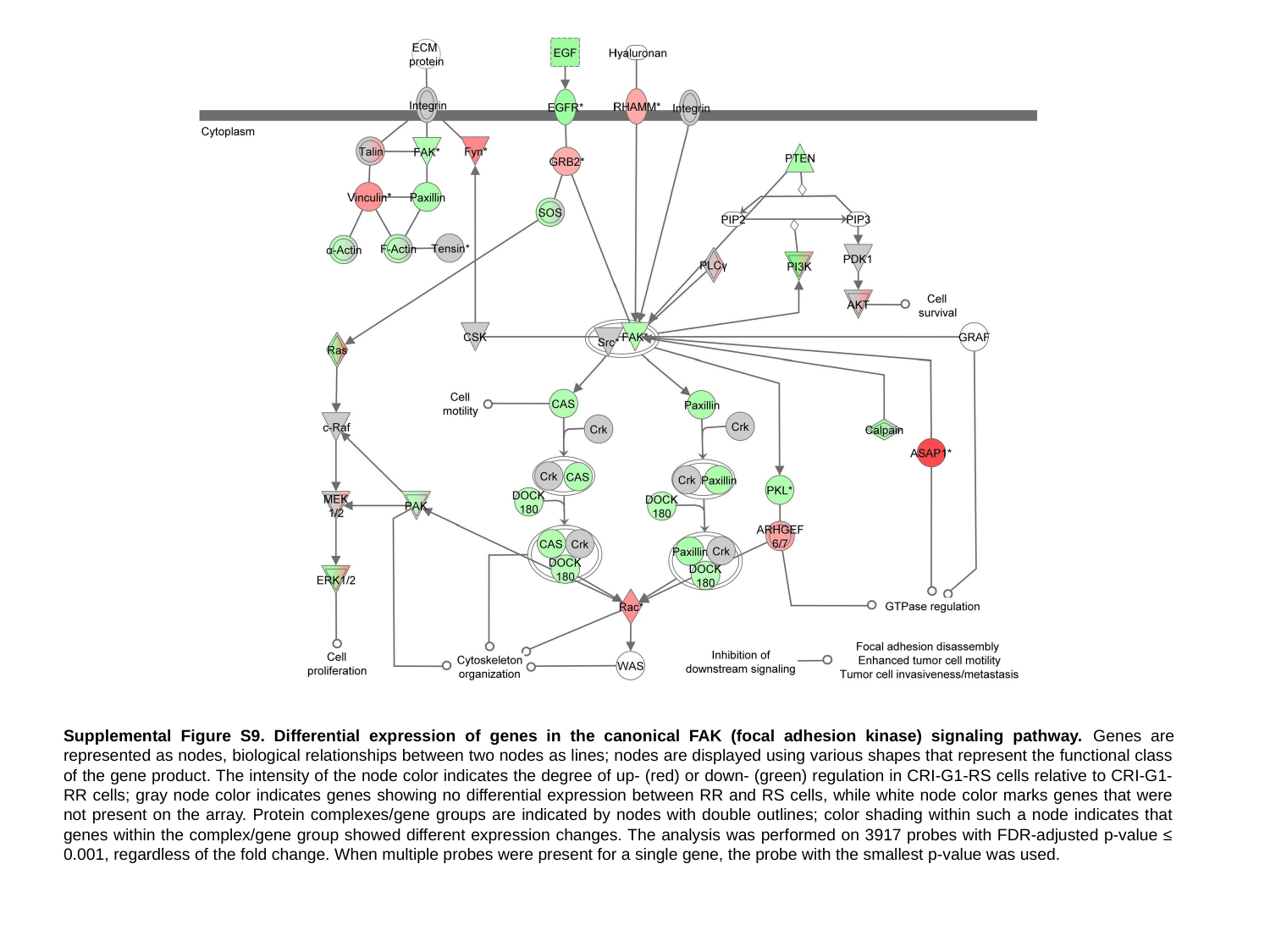

# Supplemental Figure S9. Differential expression of genes in the canonical FAK (focal adhesion kinase) signaling pathway. Genes are represented as nodes, biological relationships between two nodes as lines; nodes are displayed using various shapes that represent the functional class of the gene product. The intensity of the node color indicates the degree of up- (red) or down- (green) regulation in CRI-G1-RS cells relative to CRI-G1-RR cells; gray node color indicates genes showing no differential expression between RR and RS cells, while white node color marks genes that were not present on the array. Protein complexes/gene groups are indicated by nodes with double outlines; color shading within such a node indicates that genes within the complex/gene group showed different expression changes. The analysis was performed on 3917 probes with FDR-adjusted p-value ≤ 0.001, regardless of the fold change. When multiple probes were present for a single gene, the probe with the smallest p-value was used.
