# Supplementary material for: Molecular Basis for Vulnerability to Mitochondrial and Oxidative Stress in a Neuroendocrine CRI-G1 Cell Line
Source: PLoS One. 2011 Jan 4;6(1):e14485. doi: 10.1371/journal.pone.0014485 (PMC3020905; doi:10.1371/journal.pone.0014485)
Supplement: Figure S10 — (0.08 MB PPT) [file pone.0014485.s010.ppt]

## Slide 1
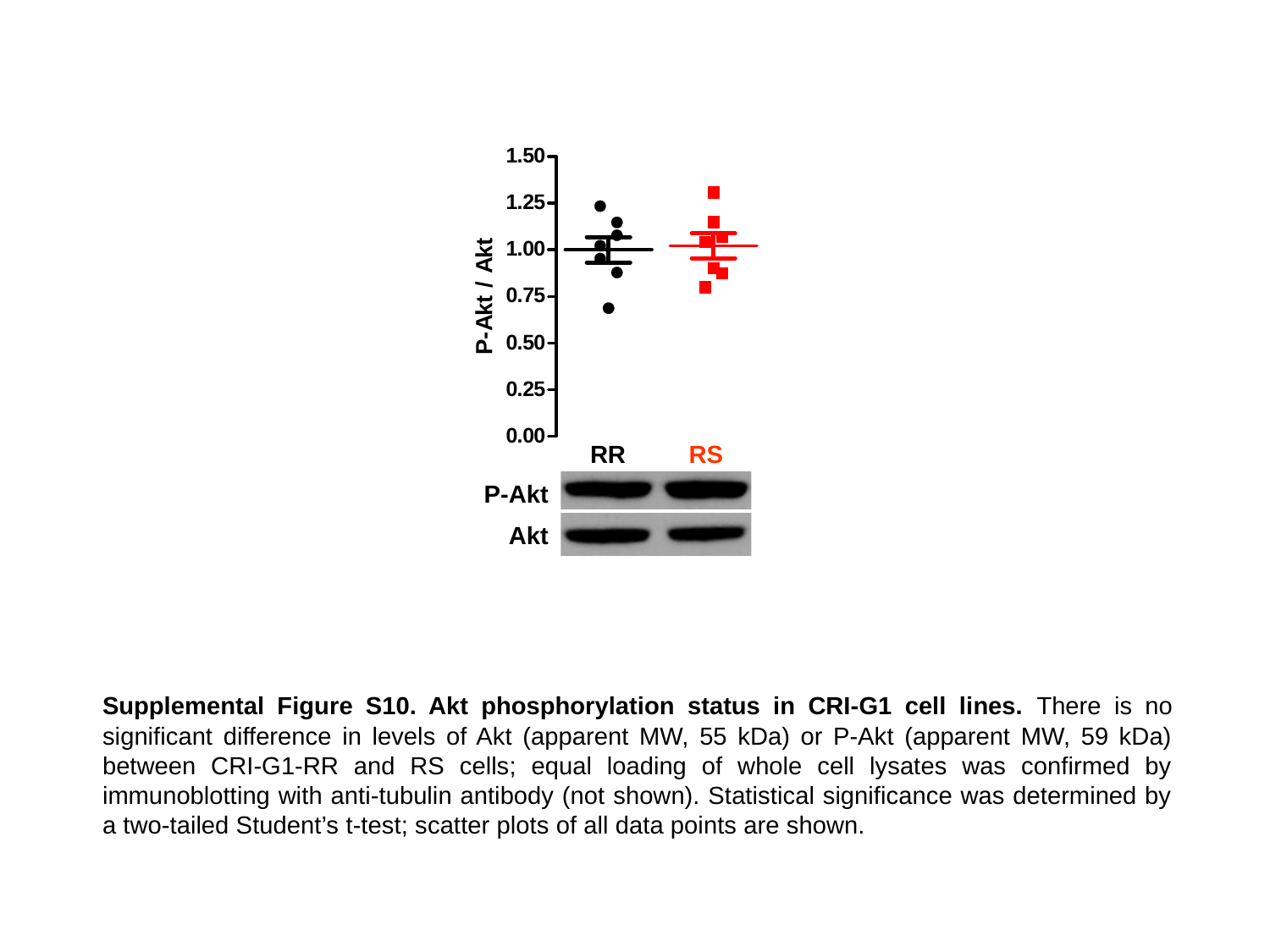

RR
RS
P-Akt
Akt
	Supplemental Figure S10. Akt phosphorylation status in CRI-G1 cell lines. There is no significant difference in levels of Akt (apparent MW, 55 kDa) or P-Akt (apparent MW, 59 kDa) between CRI-G1-RR and RS cells; equal loading of whole cell lysates was confirmed by immunoblotting with anti-tubulin antibody (not shown). Statistical significance was determined by a two-tailed Student’s t-test; scatter plots of all data points are shown.
